# Can Targeting Non-Contiguous V-Regions With Paired-End Sequencing Improve 16S rRNA-Based Taxonomic Resolution of Microbiomes?: An *In Silico* Evaluation

**DOI:** 10.3389/fgene.2019.00653

**Published:** 2019-07-12

**Authors:** Nishal Kumar Pinna, Anirban Dutta, Mohammed Monzoorul Haque, Sharmila S. Mande

**Affiliations:** Bio-Sciences R&D Division, TCS Research, Tata Consultancy Services Ltd., Pune, Maharashtra, India

**Keywords:** metagenomics 16S, paired-end sequencing, taxonomic profiling, microbiome analysis, amplicon sequencing

## Abstract

**Background:** Next-generation sequencing (NGS) technologies have enabled probing of microbial diversity in different environmental niches with unprecedented sequencing depth. However, due to read-length limitations of popular NGS technologies, 16S amplicon sequencing-based microbiome studies rely on targeting short stretches of the 16S rRNA gene encompassing a selection of variable (V) regions. In most cases, such a short stretch constitutes a single V-region or a couple of V-regions placed adjacent to each other on the 16S rRNA gene. Given that different V-regions have different resolving ability with respect to various taxonomic groups, selecting the optimal V-region (or a combination thereof) remains a challenge.

**Methods:** The accuracy of taxonomic profiles generated from sequences encompassing 1) individual V-regions, 2) adjacent V-regions, and 3) pairs of non-contiguous V-regions were assessed and compared. Subsequently, the discriminating capability of different V-regions with respect to different taxonomic lineages was assessed. The possibility of using paired-end sequencing protocols to target combinations of non-adjacent V-regions was finally evaluated with respect to the utility of such an experimental design in providing improved taxonomic resolution.

**Results:** Extensive validation with simulated microbiome datasets mimicking different environmental and host-associated microbiome samples suggest that targeting certain combinations of non-contiguously placed V-regions might yield better taxonomic classification accuracy compared to conventional 16S amplicon sequencing targets. This work also puts forward a novel *in silico* combinatorial strategy that enables creation of consensus taxonomic profiles from experiments targeting multiple pair-wise combinations of V-regions to improve accuracy in taxonomic classification.

**Conclusion:** The study suggests that targeting non-contiguous V-regions with paired-end sequencing can improve 16S rRNA–based taxonomic resolution of microbiomes. Furthermore, employing the novel *in silico* combinatorial strategy can improve taxonomic classification without any significant additional experimental costs and/or efforts. The empirical observations obtained can potentially serve as a guideline for future 16S microbiome studies, and facilitate researchers in choosing the optimal combination of V-regions for a specific experiment/sampled environment.

## Introduction

Sequencing of 16S rRNA genes is a standard protocol for taxonomic characterization of bacterial species (Schmalenberger et al., 2001; Clarridge, 2004; Munson et al., 2004; Petti et al., 2005). Sanger sequencing has been conventionally used for obtaining “full-length” 16S rRNA gene sequences of individual bacterium. Advent of next-generation sequencing (NGS) platforms has empowered the field of metagenomics and has enabled one to amplify and sequence (amplicon sequencing) specific portions of the 16S rRNA gene of community of bacteria (microbiome). Sequencing of such regions (encompassing one or more variable regions or V-regions) has been utilized in microbiome studies for obtaining taxonomic assignments for bacterial groups present in the studied environment. Although the accuracy and depth of taxonomic attribution obtained using such short reads are not at par as compared to that obtained using longer reads (Soergel et al., 2012; Martínez-Porchas et al., 2016), adoption of the former approach allows sequencing/sampling of large volumes of environmental DNA at significantly lower costs (Liu et al., 2012).

Depending on the sequencing platforms used, microbiome studies utilize either a single variable (V) region or a stretch of V-regions. For example, some of the Illumina platforms which generate very short reads (∼150–250 base pairs in length) can be used to target only a single V-region using fragment library sequencing protocol (Bartram et al., 2011). On the other hand, technologies like Ion Torrent, Roche 454 etc., can generate longer reads (∼400–500 bp) encompassing 2 or 3 contiguously placed V-regions (Loman et al., 2012; Salipante et al., 2014; D’Amore et al., 2016; Panek et al., 2018). Similar longer reads may also be targeted using a paired-end sequencing protocol on Illumina platforms (Fadrosh et al., 2014). It may also be noted that paired-end sequencing protocols, in principle, allows targeting and sequencing two sufficiently separated (non-contiguous) variable regions located on the same 16S rRNA gene (by choosing appropriate primers). Although paired-end sequencing has been in use for quite a while and have been used for whole-genome shotgun (WGS) sequencing-based metagenomics studies (Feng et al., 2015; Moustafa et al., 2018), to our knowledge, none of the 16S rRNA-based microbiome profiling studies have targeted or utilized a combination of “non-contiguous” V-regions for taxonomic characterization of bacterial communities. A few earlier studies have examined different aspects of short-read sequencing study designs with the goal of optimizing the choice of sequencing protocol (single-end *vs*. paired-end), target V-regions, as well as the taxonomic classification algorithm (Zhang et al., 2018; Yadav et al., 2019). A recent study has also attempted to combine taxonomic information from multiple V-regions (Fuks et al., 2018). Given the variable utility of different V-regions in resolving different bacterial taxonomic groups, it is also pertinent to ask whether the choice of V-regions should be restricted to a contiguous stretch, or be extended to a combination of V-regions placed “non-contiguously.” To probe this at depth, we have performed comparison of taxonomic classifications obtained using various V-regions and their combinations. We have also assessed the feasibility of using “non-contiguous” V-region combinations for obtaining an accurate (and relatively higher resolution) taxonomic profile of a microbiome. The accuracy of taxonomic classifications obtained (at various levels of taxonomic hierarchy) using such non-contiguous V-regions has been compared with those obtained using single V-regions as well as with conventionally used combinations of contiguous V-regions.

## Methods

The primary objective of the current study involves evaluating/comparing the accuracy of taxonomic profiles generated from sequences encompassing (a) individual V-regions, (b) adjacent V-regions, and (c) pairs of non-contiguous V-regions and further assessing the discriminating capability of different V-regions with respect to different taxonomic lineages.

Full-length bacterial 16S rRNA gene sequences (along with their annotated lineages) present in the RDP database (release 11.3) (Cole et al., 2014) were downloaded for different analyses (described later in this section) in view of the abovementioned objectives. The RDP hierarchy browser (https://rdp.cme.msu.edu/hierarchy/hb_intro.jsp) was used for this purpose with the following filters—strain = “both”; source = “isolates”; size “> = 1,200”; taxonomy = “NCBI”; quality = “good,” which resulted in a downloaded set of 232,163 sequences. Further, sequences not containing any of the nine V-regions (V1–V9) were filtered out from the set of sequences, leaving a total of 84,711 16S rRNA sequences belonging to 11,810 species, all of which contained all nine V-regions. Subsequently, both full-length as well as different portions of the 16S rRNA gene sequences were extracted *in silico* to represent outcomes of amplicon sequencing experiments and were provided as input to the Wang classifier (algorithm used in RDP classifier), as implemented in the software Mothur v.1.29.2 (Schloss et al., 2009), for taxonomic classification. The current version of RDP classifier 16S training set (https://sourceforge.net/projects/rdp-classifier/files/RDP_Classifier_TrainingData/RDPClassifier_16S_trainsetNo16_rawtrainingdata.zip/download) was used as the reference database for these taxonomic assignment steps, and the taxonomic hierarchy information of the reference sequences were appropriately used while training the Wang classifier in order to enable obtaining taxonomic classifications resolved up to species level. Only a subset (57,632 sequences) of the originally downloaded full-length 16S rRNA gene sequences, which could be classified at species level with > = 80% bootstrap confidence threshold, was later used as a pool for randomly drawing sequences during creation of mock/simulated microbiome datasets (as described later in this section).

While evaluating the discriminating ability of individual V-regions, the regions of interest were parsed out from corresponding full-length 16S rRNA gene sequences using an in-house modified version of the V-Xtractor program (Hartmann et al., 2010), and submitted as query sequences to the Wang classifier. It may be noted in this context that reads generated during amplicon sequencing may often encompass flanking “constant” regions in addition to the targeted V-region(s), depending on choice of primers and the maximum read-length attainable by the sequencing technology. Consequently, our evaluation exercise, pertaining to combination of V-regions, aimed at mimicking 250 bp × 2 paired-end sequencing, wherein the extracted regions (representing sequenced reads) also encompass such flanking regions. To achieve this, regions from the full length 16S rRNA genes were extracted in such a way that either of the 250 bp reads (constituting a read-pair) contained one of the target V-regions, flanked in both directions by certain portions (lengths) of the surrounding “constant” regions. HMMs corresponding to constant regions surrounding the V-regions, as provided by the V-Xtractor program, were used for this purpose. Each extracted read started from a selected HMM near the target V-region (akin to a sequencing primer) and was extended to up to 250 bp toward the direction of the target V-region, thereby creating a read which encompassed the V-region along with some flanking sequence portion. It may be noted here that actual primer design may not always allow retention of flanks on either side of the targeted V-regions, equivalent to what was obtained using the HMMs, and results from an actual sequencing experiment may therefore slightly vary from the *in silico* validation results presented in this work. In case two adjacent V-regions were targeted, there was a significant chance of finding an overlap between two reads constituting a pair. This overlap was utilized to join the pair of reads together (used the program PEAR v0.9.6 with default parameters) (Zhang et al., 2014) into a single sequence before submitting the same as a query to the Wang classifier. In contrast, on targeting two distantly separated non-contiguous V-regions, no overlap between the read pairs could be expected. Accordingly, the pair of reads in this case were concatenated using a string of eight consecutive “*N*s,” while preserving their orientation, prior to processing with Wang classifier. Given that Wang classifier (or RDP classifier) utilizes 8-mer nucleotide frequencies during taxonomic assignment (Wang et al., 2007), joining two non-overlapping sequenced fragments with 8 ambiguous nucleotides (N) ensures avoiding generation of spurious 8-mers consisting nucleotides from nonadjacent regions of the gene. The merging and concatenating of paired-end reads is depicted in a schematic diagram provided in **Supplementary Figure S1**. Taxonomic assignments generated by the Wang classifier at a predetermined taxonomic level with a confidence threshold score of > = 80% were used for all downstream comparative analyses. The different analyses performed and the underlying rationales are described in the following paragraphs.

First, the effectiveness of individual V-regions in resolving between different taxonomic groups was evaluated. For this purpose, different V-regions from all the 16S rRNA gene sequences, downloaded from the RDP database, were extracted. Subsequently, each of these individual V-regions were subjected to taxonomic classification with the Wang classifier (Wang et al., 2007), and the resultant assignments at the genus level were checked for accuracy and specificity against the taxonomic attributes provided by RDP for the corresponding full-length sequences.

The utility of all possible pair-wise combinations of V-regions, either arranged contiguously or non-contiguously, was also investigated *in silico* in terms of accuracy of taxonomic classifications provided by each such combination. As mentioned earlier, sequence fragments mimicking outcomes of 250 bp x 2 paired-end sequencing, which target different contiguous/non-contiguous combinations of V-regions, were derived from the downloaded 16S rRNA gene sequences. These fragments were subsequently subjected to taxonomic classification with the Wang classifier (Wang et al., 2007), and the assignments obtained at species level were checked for accuracy and specificity against the pre-annotated taxonomic attributes of their source (full-length) 16S rRNA genes.

The specific combinations of V-regions, which provided comparatively higher accuracies of taxonomic classification with the RDP database sequences, were further evaluated in a taxonomic assignment exercise with mock microbiome datasets. Five mock 16S microbiome gene pools were created from randomly selected sets of 50 organisms (genera) listed in RDP database (**Supplementary Table S1**). To obtain reads for building the mock microbiome datasets corresponding to these pools, each time, 10,000 16S rRNA genes were drawn randomly (following a uniform distribution) from a gene pool, such that the proportion of 16S rRNA genes drawn from any of the organisms are also randomized. Five such datasets (with 10,000 reads each) corresponding to each of the five gene pools (a total of 25 mock datasets) were constructed for comparative evaluation. Different contiguous as well as non-contiguous combinations of V-regions were subsequently extracted from each of the 16S rRNA genes belonging to these mock datasets and subjected to taxonomic analysis using Wang classifier, following the classification methodology described above. Taxonomic abundance values (obtained using different combinations of V-regions) were averaged over five mock datasets pertaining to the same gene pool. The averaged abundance values for each of the mock gene pools were compared against each other and the pre-annotated taxonomic attributes of their source (full-length) 16S rRNA genes, to assess the utility of the chosen combinations of V-regions. Nine more simulated microbiomes mimicking different environmental and host associated niches—namely, gut, skin, vaginal, sub-gingival (oral), sputum (oral), nematode gut, soil, and aquatic were also generated. Taxonomic abundance estimates for eight of these environmental microbiomes were derived from datasets used in an earlier *in silico* study evaluating functional potential of diverse metagenomes (Nagpal et al., 2016). Taxonomic abundance estimates for the aquatic microbiome was derived from a recent study by Muscarella and co-workers (Muscarella et al., 2019). To populate these simulated microbiomes, sequences from RDP database were randomly drawn (exact distributions provided in **Supplementary Table S2**), while making sure that the proportions of 16S rRNA genes drawn from different genera were roughly similar to the proportions observed earlier for these environments (Cui et al., 2012; Griffen et al., 2012; Human Microbiome Project Consortium, 2012; Alekseyenko et al., 2013; Botero et al., 2014; Kato et al., 2014; Romero et al., 2014; Xiao et al., 2014; Muscarella et al., 2019) (**Supplementary Table S3**). The taxonomic classification efficiency of the V-region combinations (at the species level) was also assessed on this set of simulated microbiomes.

**Figure 1:**
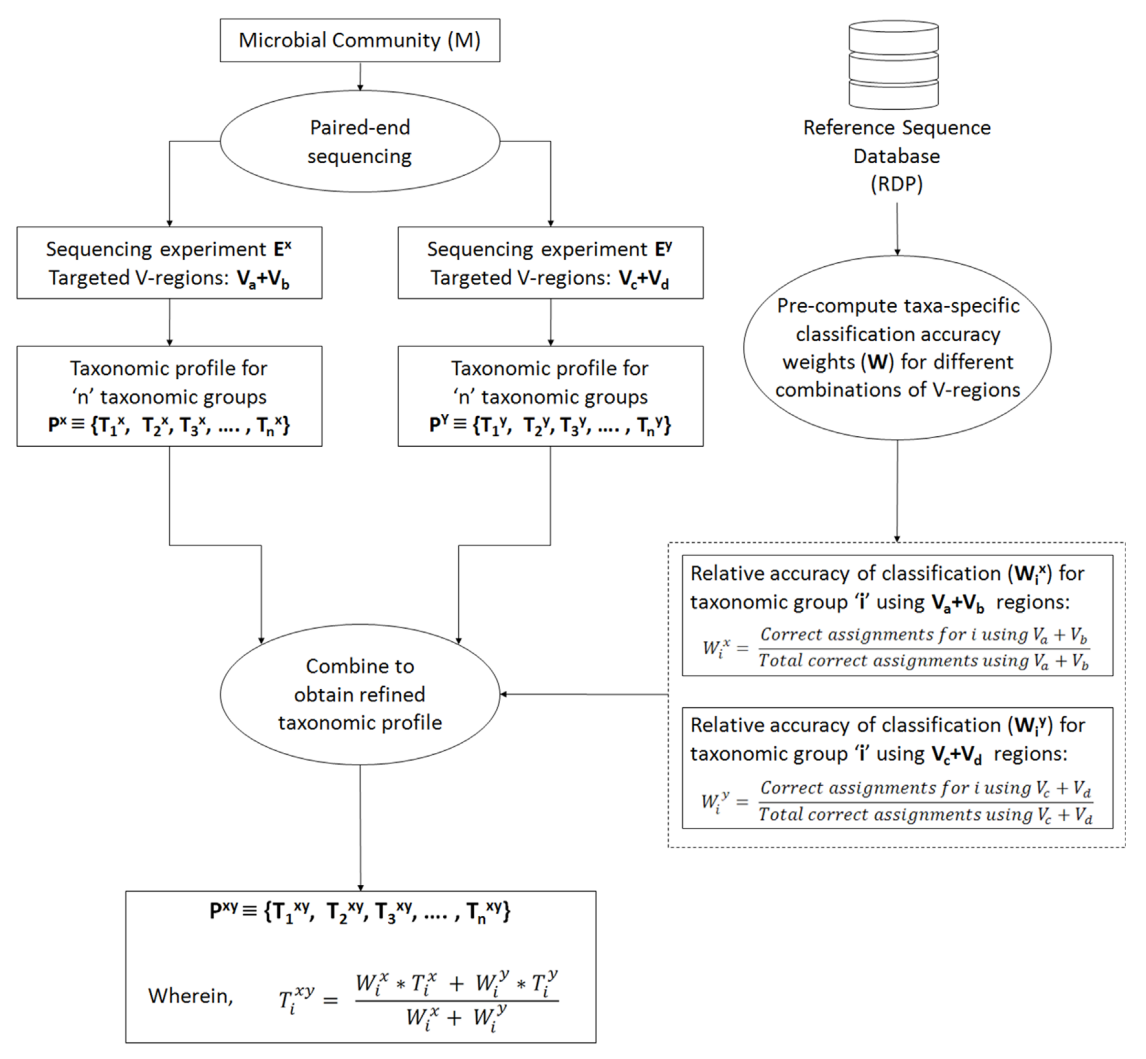
Combinatorial strategy for targeting multiple pair-wise combinations of non-contiguous (or contiguous) V-regions. The strategy relies on obtaining taxonomic abundance profiles of a microbial community from two paired-end sequencing experiments, each of which targets different pair-wise combinations of V-regions. The two taxonomic profiles are then combined based on the pre-calculated accuracies of individual V-regions (targeted in the two experiments) in resolving each of the taxonomic groups under consideration.

In an ideal scenario, better taxonomic classification accuracy can be aimed for by using information from multiple V-regions. However, due to experimental limitations, this can be attained only if a long-read sequencing technology is used. To overcome this limitation, we propose a combinatorial strategy that extends the described paired-end sequencing workflow for targeting multiple pair-wise combinations of non-contiguous (or contiguous) V-regions in the following manner. The proposed strategy relies on obtaining taxonomic abundance profiles of a microbial community from two paired-end sequencing experiments, each of which targets different pair-wise combinations of V-regions. The two taxonomic profiles are then combined based on the accuracies of the individual V-regions (targeted in the experiments) in resolving each of the taxonomic groups under consideration. **Figure 1** and the following generic example illustrate the strategy in detail: A microbial community (M) is initially considered for taxonomic profiling by two paired-end sequencing experiments (E^x^ and E^y^). Each of these experiments can target two distinct V-regions (either arranged contiguously or non-contiguously on the 16S rRNA gene), using appropriate forward and reverse primers, as described in the previous sections. Let us consider that in the current example, E^x^ targets the V-region combination V_a_+V_b_, and E^y^ targets V_c_+V_d_. For example, combinations of V-regions selected in the two experiments could be V1+V4 and V2+V6 in one scenario. Based on the taxonomic resolution efficiencies of different (combinations of) V-regions, E_x_ and E_y_ will generate two different taxonomic abundance profiles P^x^ and P^y^, respectively, each of which constitutes of estimated abundance values (T_i_) for different taxonomic groups (i):

Equation 1 $${\text{P}}^{\text{x}}\equiv \{T_{1}^{\text{x}},T_{2}^{\text{x}},T_{3}^{\text{x}},\ldots \ldots ,T_{\text{n}}^{\text{x}}\}$$

Equation 2 $${\text{P}}^{\text{x}}\equiv \{T_{1}^{\text{y}},T_{2}^{\text{y}},T_{3}^{\text{y}},\ldots \ldots ,T_{\text{n}}^{\text{y}}\}$$

Subsequently, for each of the taxonomic groups (T_i_), a refined estimate of its abundance (T_i_^xy^) can be arrived at by combining the observed abundances T_i_^x^ and T_i_^y^, such that the refined abundance T_i_^xy^ is relatively closer to the estimate obtained with the experiment (either of E^x^ or E^y^) providing better classification accuracies for taxa ‘i.’ Calculation of the refined estimate therefore takes into consideration the taxonomic classification accuracies of the combination of V-regions that had been used for the initial set of experiments E^x^ and E^y^ using the following equation:

Equation 3 $$T_{i}^{xy}=\;\;\frac{W_{i}^{x}*T_{i}^{x}\;+\;W_{i}^{y}*T_{i}^{y}}{W_{i}^{x}+\;W_{i}^{y}}$$

wherein W_i_^x^ and W_i_^y^ are the relative accuracies in taxonomic classification for a particular taxonomic group ‘i,’ obtained using the specific combination of V-regions chosen for experiments E^x^ and E^y^ respectively. In case the refined taxonomic profiles are to be represented in terms of normalized abundance values, e.g., frequencies or percentage normalized abundances, the refined T_i_^xy^ values from equation 3 needs to be appropriately modified (normalized) further. This weighted average approach has been adopted considering that different V-regions (or their combinations) have different efficiencies in resolving the same taxonomic group. A simple average therefore would not be appropriate for combining two taxonomic abundance estimates pertaining to a sample, which has been generated through separate experiments targeting different V-regions (or their combinations). Instead, the refined taxonomic abundance value for a given taxon should be weighted toward the results generated by the V-region (or a combination) which is more accurate in classifying the taxon in question. These accuracies can be calculated from the evaluation results obtained from **Supplementary Table S4**, as a ratio of the correct assignments obtained for particular taxa using a specific combination of V-regions, and the total number of correct assignments obtained using the same V-region combination. For example, considering that the combination of V_a_+V_b_ was used in experiment E^x^, W_i_^x^ can be calculated as

Equation 4 $$W_{i}^{x}=\;\frac{Correct\;assignments\;for\;taxon\;i\;using\;V_{a}+V_{b}}{Total\;correct\;assignments\;using\;V_{a}+V_{b}}$$

The denominator term representing “total correct assignments using V_a_+V_b_” has been introduced to capture any additional specificity of the chosen V_a_+V_b_ region toward a particular taxon ‘i’ in context of the overall taxonomic classification performance of V_a_+V_b_. Other simple ways of calculating the “relative accuracy in taxonomic classification” or weight (W_i_^x^), e.g., in a case wherein the denominator term is omitted, would also work fine when V-region combinations with decent classification accuracy are chosen. It may be noted here that in the experiment(s) using paired-end sequencing to capture two different V-regions from the 16S rRNA gene, the correspondence between the pairs of V-regions originating from the same 16S rRNA gene is retained. This allows joining the different V-regions together into a single DNA string (separated appropriately by ambiguous nucleotide characters) and providing the same as an input to taxonomic classification tools, such as the RDP classifier. However, for V-regions targeted in separate sequencing experiments, cross-experiment correspondence between the sequenced V-regions with respect to their origin 16S rRNA gene cannot be identified. This necessitates the indirect strategy of combining information obtained from different V-regions (or their combinations) for refining the taxonomic abundance estimates, as described above. To avoid variations arising from experimental workflows and sample handling/preparations, it would be ideal to perform a single PCR step for amplicon generation, using different sets of primers appropriate for the chosen combinations of V-regions (V_a_+V_b_, and V_c_+V_d_ in the given example). However, it also needs to be mentioned here that the designed primers may have different affinities for the targeted regions on 16S rRNA genes originating from different taxonomic groups. This may again result in unequal proportions of 16S rRNA sequence fragments amplified by the different sets of primers, which would subsequently be reflected in the sequencing outcome. In such a scenario, the combination strategy needs to factor in this difference in proportions, while arriving at a refined taxonomic abundance estimate. Alternately, the experiment may target a combination of 3 V-regions (e.g., V_a_+V_b_ and V_a_+V_c_ or, V_a_+V_c_ and V_b_+V_c_), such that either the forward primers or the reverse primers be common to the targeted combinations. This way, some equivalence in the proportions of fragments (targeting different taxonomic groups) can be maintained on account of the shared primer (for V-region) selected.

To asses the utility of the combinatorial strategy, the taxonomic abundance profile of the simulated microbiome sample pertaining to human gut (as described earlier) was re-evaluated, targeting the V-region combinations V_1_+V_4_ and V_1_+V_5_, both of which had decent classification accuracies. 5,000 sequence fragments corresponding to each of the V-region combinations (i.e., a total of 10,000 fragments) were sampled from the simulated gut microbiome. The results obtained with the combinatorial strategy was subsequently compared against the results obtained when each of the V-region combinations were used separately. To maintain equivalence in sequencing coverage, 10,000 fragments were sampled from the simulated gut microbiome, while targeting the V-region combinations separately.

## Results and Discussion

### Individual V-Regions Have Differential Ability in Resolving Various Taxonomic Groups

**Figure 2:**
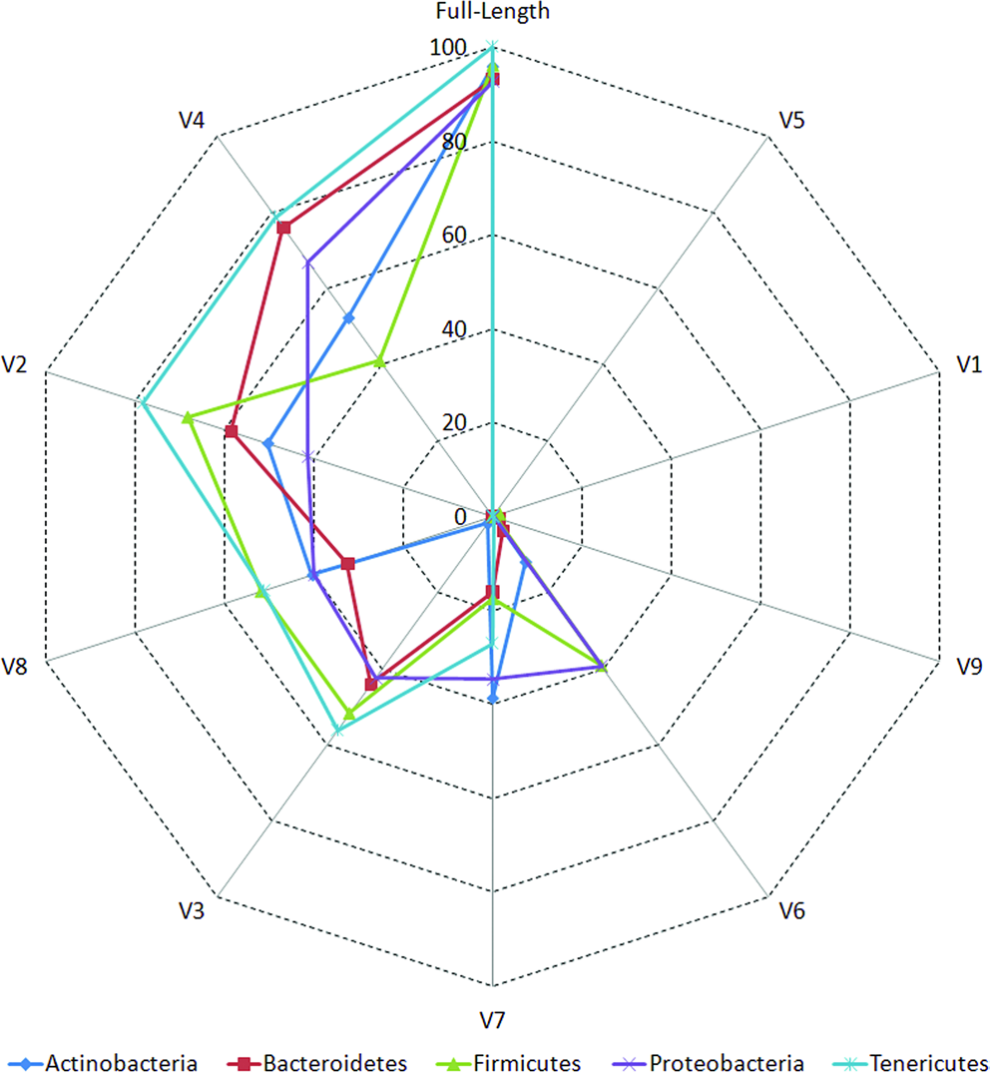
Taxonomic classification accuracies at genus level for different variable regions. Plot depicting the percentage of 16S rRNA genes present in RDP database that could be correctly classified utilizing different variable (V) regions (see Methods). Correct classifications obtained using full-length 16S sequences are also depicted for comparison. Taxonomic classification accuracy at genus level has been considered in this plot and has been cumulated and depicted at the phylum level (only for five most represented phyla in the downloaded RDP sequences).

The accuracies of different V-regions in resolving different taxonomic groups are depicted in **Figure 2**. The classification accuracies (at genus level) obtained with V-regions have been cumulated and depicted at the “phylum level” in the figure and placed in context with the classification accuracies which would have been obtained with full-length 16S rRNA gene sequences (details in Methods). Except for V1, V5, and V9, all other V-regions were observed to have certain utility in taxonomic classification, even when targeted individually. It was also evident from the plot that some V-regions provide comparatively higher accuracies of classification for specific taxonomic groups. For example, the V4 region has the highest accuracy while classifying sequences pertaining to the phylum *Bacteroidetes* (75.9%), whereas the V2 region classifies best with respect to the phylum *Firmicutes* (68.2%). However, it may be noted that a sequenced read generated in a real amplicon sequencing experiment will extend beyond the targeted V-regions and include some surrounding portions. The resultant taxonomic classification in such a case is expected to be better than the currently depicted results which were generated based on the exact V-regions. A detailed list of accuracies in taxonomic classification obtained with different V-regions at genus level is provided in **Supplementary Table S5**. Given these observations, it would seem logical for a microbiome study design to sequence two (or more) V-regions from a 16S rRNA gene fragment which have complementary abilities with respect to classification of different taxonomic groups. Furthermore, the choice of the combination of V-regions could also be guided by the environment from where the microbiome sample is being collected, given that diverse environments may be differentially enriched with different taxonomic groups.

A preferred combination of V-regions cannot always be expected to be situated in a contiguous stretch on the 16S rRNA gene. Given the read length limitations of NGS technologies, targeting an amplicon constituting the preferred regions becomes difficult in reality. The length distributions of V-regions and C-regions (constant/conserved regions flanking the V-regions) across different bacterial taxonomic groups are provided in **Supplementary Figure S2**. These distributions indicate that while individual V-regions and contiguous stretches like V2–V3 (median length 297 bp) or V3–V4 (median length 254 bp) can easily be targeted with short-read sequencing techniques like Illumina HiSeq/MiSeq, sequencing longer contiguous stretches encompassing more than two V-regions, such as V2-V3-V4 (median length 482 bp) and V4-V5-V6 (median length 453 bp), necessitates sequencing platforms that can generate longer read lengths (e.g., Roche 454). Capturing even more V-regions on a single read is beyond the scope of most current generation high-throughput sequencing technologies. Consequently, targeting an optimal combination of V-regions, which may be present on the 16S rRNA gene in either contiguous or non-contiguous arrangement(s), remains a challenge.

### Targeting Combinations of Non-Contiguously Placed V-Regions Using Paired-End Sequencing Enables Improved Taxonomic Classification

Paired-end sequencing protocols available with some of the NGS platforms allow sequencing of a stretch of DNA from both its ends (Rodrigue et al., 2010; Dutta et al., 2014). For example, Illumina HiSeq sequencing platforms can be used for paired-end sequencing to generate up to 2x250bp reads. The current work proposes, and evaluates *in silico*, the utilization of paired-end sequencing protocols for sequencing various pair-wise combinations of non-contiguous V-regions in a single sequencing run. To this end, appropriate primers need to be designed against a desired stretch of the 16S rRNA gene, such that the targeted V-regions (either contiguously or non-contiguously placed) reside within this stretch and are not far from either of its boundaries. Sequencing of the amplicons generated with these primers can then be performed with a paired-end sequencing protocol, whereby these (amplified) stretches of DNA are sequenced from both ends. Two reads sequenced from each such amplicon would cover the two targeted V-regions (one from each end). Since each of the sequenced reads from any given “pair” targets a single V-region (situated at one of the ends of the amplicon), read-length limitations do not restrict capturing the entirety of the individual V-regions. Consequently, it becomes possible to sequence almost all possible pair-wise combinations of V-regions, either arranged contiguously or non-contiguously.

**Figure 3:**
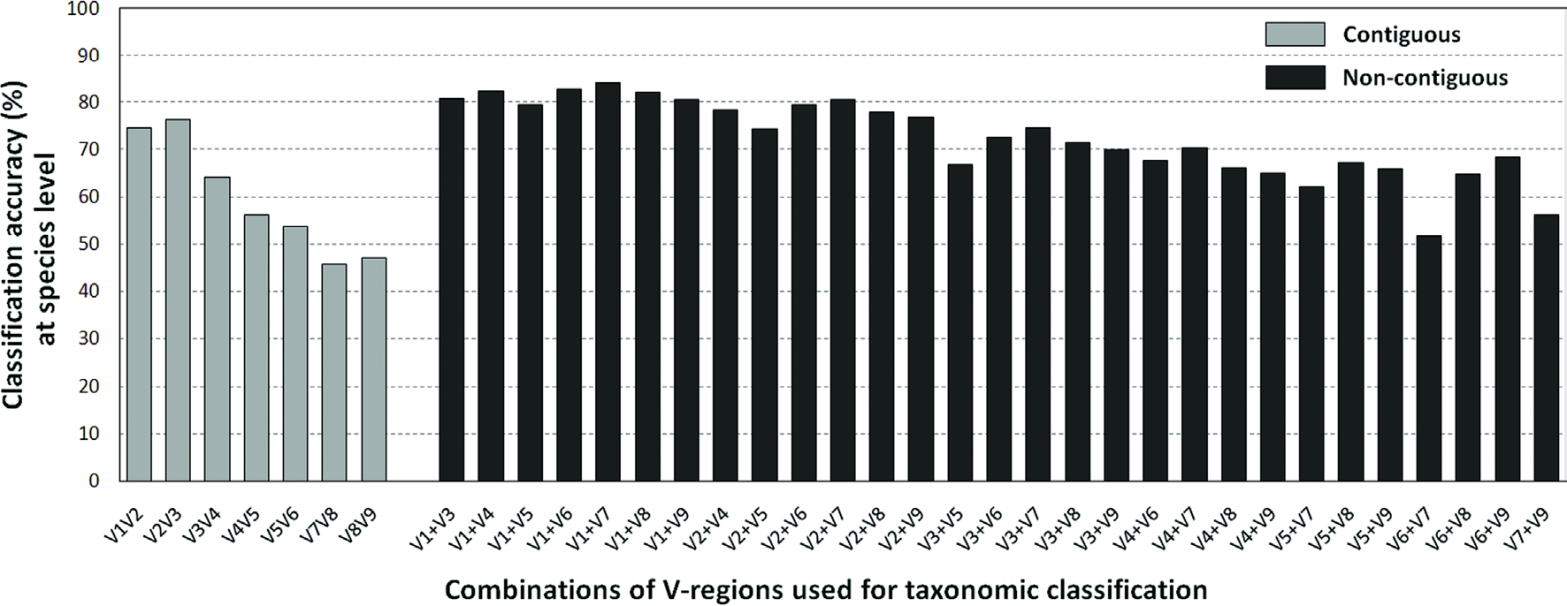
Taxonomic classification accuracies at species level for different variable regions. Plot depicting the average taxonomic classification accuracies obtained at species level using different pair-wise combinations of V-regions (both contiguous as well as non-contiguous) drawn from the 16S rRNA genes. 16S rRNA genes used for the evaluation were retrieved from the RDP database (see Methods).

The results pertaining to the *in silico* evaluation of the effectiveness of different combinations of V-regions (see Methods), in providing accurate taxonomic classifications (at the species level) for sequences listed in the RDP database, is depicted in **Figure 3** (also see **Supplementary Table S4**).

**Figure 4:**
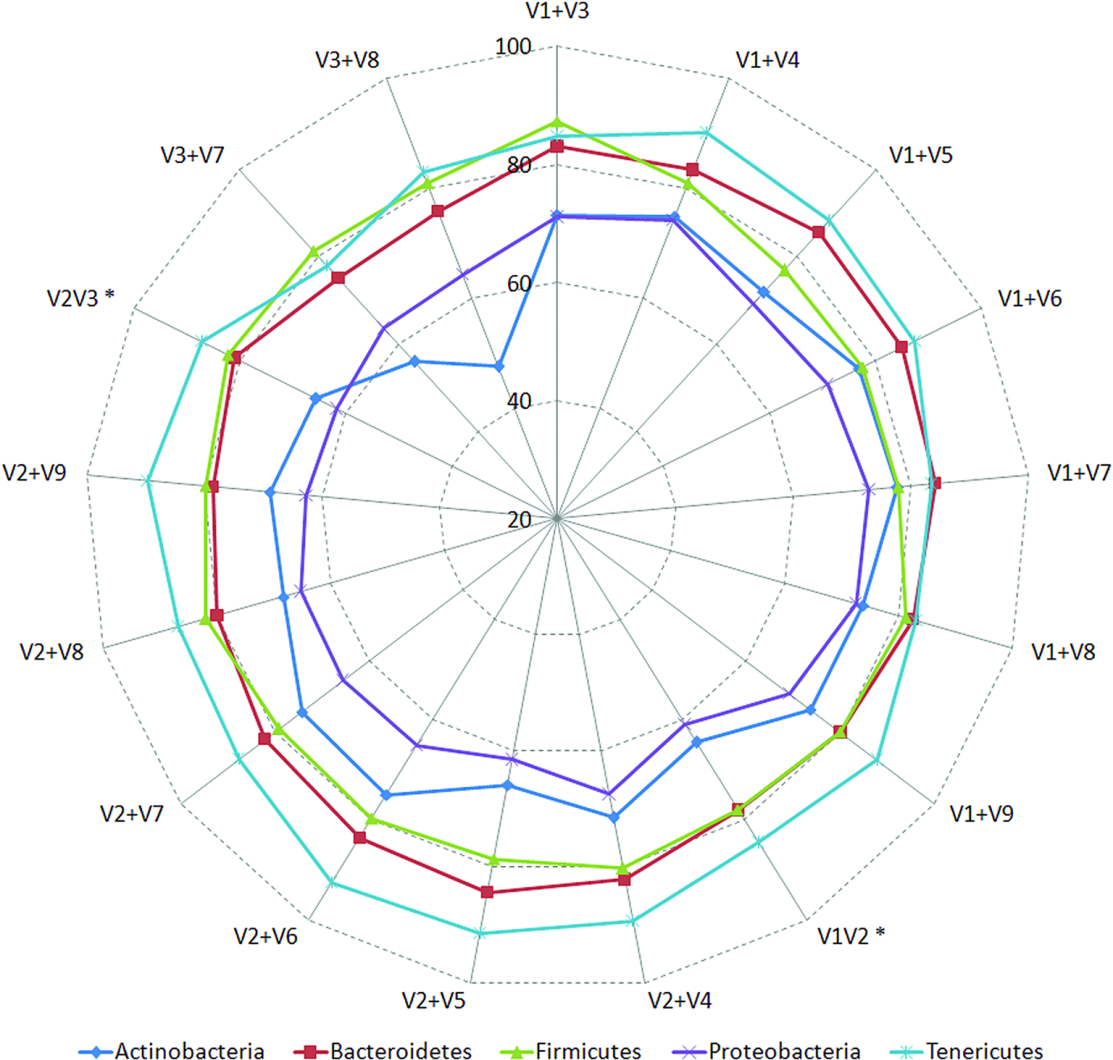
Taxonomic classification accuracies obtained using different pair-wise combinations of V-regions (contiguous as well as non-contiguous). Accuracy of taxonomic assignments has been evaluated at the species level and cumulated at phylum level for representation (only for five most represented phyla in the downloaded RDP sequences). Combinations of V-regions achieving a classification accuracy of > = 70% (averaged for the depicted phyla) are shown. Combinations of contiguously placed V-regions have been indicated with an asterisk (*).

Classification accuracies provided by several combinations of non-contiguously placed V-region pairs, namely, V1+V3 (77.7%), V1+V4 (77.4%), V1+V8 (76.6%), V2+V5 (73.6%), etc., were sufficiently high and exceeded the classification accuracies provided by even the best of the combinations of adjacently placed V-regions (e.g., 68.6% by V1+V2, 70.9% by V2+V3) by a fair margin of 5–8%. It was also significant to note that many of the individual V-regions, which had very low taxonomic discriminating ability of their own (**Figure 2**, **Supplementary Table S5**), could provide significant classification accuracies when paired up with other V-regions. For example, while V1 and V5 provided very low taxonomic classification accuracies when targeted alone, the combination of V1+V5 could provide a significantly high taxonomic classification accuracy of 73.4%. Furthermore, although the individual V-regions were observed to have differential abilities in classifying sequences originating from different phyla (**Figure 2**), their combinations were much more coherent in this regard and could classify sequences from all phyla with better efficiency (**Figure 4**) than single V-regions. Results indicate the potential utility of targeting pairs of non-contiguously placed V-regions to improve taxonomic classification accuracy. Additionally, the results also suggest that for exploring the taxonomic diversity of a particular environment, which may be expected to be enriched with particular groups of bacteria, an appropriate combination of V-regions sensitive to the same bacterial groups may be chosen.

To assess the utility of the proposed non-contiguous combination of V-regions on a microbiome dataset, while avoiding any bias arising out of the proportion of sequences pertaining to different bacterial groups currently catalogued in reference databases like RDP, taxonomic classification exercises were further performed with mock microbiome datasets. Each of the mock microbiome datasets were constructed using 10,000 randomly selected 16S rRNA gene sequences from one of the five randomized 16S gene pools. Each of these gene pools consisted of sequences downloaded from the RDP database, wherein the proportion of sequences selected from different organisms were also randomized (see Methods). The results, in terms of classification accuracy at the species level, are depicted in **Table 1**. It was interesting to note that 18 out of the 20 combinations of V-regions, which could provide classification accuracy > = 60% on average, constituted of non-contiguous V-regions. The best performing combination of adjacent V-regions was V2–V3, which on average provided 69.1% classification accuracy. In comparison, the combination of the non-contiguously placed V-regions V1+V4 demonstrated a high average classification accuracy of 77.2%.

**Table 1:** Taxonomic classification accuracies obtained using different pair-wise combinations of V-regions (both contiguous as well as non-contiguous) evaluated for mock microbiome datasets, each constituting of 10,000 randomly selected 16S rRNA genes from five different 16S gene pools.

Combination of V-region	Classification accuracy (%) at species level averaged over five mock datasets from each 16S gene pool					
	Mock datasets from 16S gene pool 1	Mock datasets from 16S gene pool 2	Mock datasets from 16S gene pool 3	Mock datasets from 16S gene pool 4	Mock datasets from 16S gene pool 5	Average accuracy
V1+V4	77.29	79.47	72.79	75.90	80.48	77.19
V1+V3	74.69	78.16	77.52	74.76	80.08	77.04
V1+V8	76.03	77.96	73.24	75.72	79.32	76.46
V1+V7	77.20	78.33	70.37	77.34	78.60	76.37
V1+V6	72.46	77.34	69.73	78.25	76.90	74.94
V1+V5	70.89	74.24	69.16	73.37	76.40	72.81
V1+V9	71.74	71.41	71.33	73.95	75.57	72.80
V2+V4	69.07	75.07	72.76	70.99	73.55	72.29
V2+V8	68.26	74.60	73.33	70.66	73.27	72.02
V2+V6	66.84	74.54	72.60	72.19	72.67	71.77
V2+V7	68.34	72.76	72.73	71.17	71.30	71.26
V2V3*	61.53	71.52	72.03	66.31	73.92	69.06
V2+V9	65.03	68.85	71.60	66.32	71.81	68.72
V1V2*	64.20	70.29	66.81	65.44	72.40	67.83
V3+V8	68.47	61.80	69.66	66.59	67.82	66.87
V3+V7	68.41	61.60	71.05	66.80	65.93	66.76
V2+V5	61.38	68.19	68.42	65.36	69.34	66.54
V3+V6	63.26	59.91	68.53	67.04	65.15	64.78
V3+V9	63.63	55.85	67.20	65.94	63.83	63.29
V3+V5	60.94	56.74	65.79	62.91	62.49	61.77

Accuracy of taxonomic assignments has been evaluated at the species level considering the assignments obtained with full-length 16S sequences to be correct. Top 20 combinations in terms of average classification accuracy have been depicted. Combinations of contiguous V-regions have been marked with an asterisk (*).

**Figure 5:**
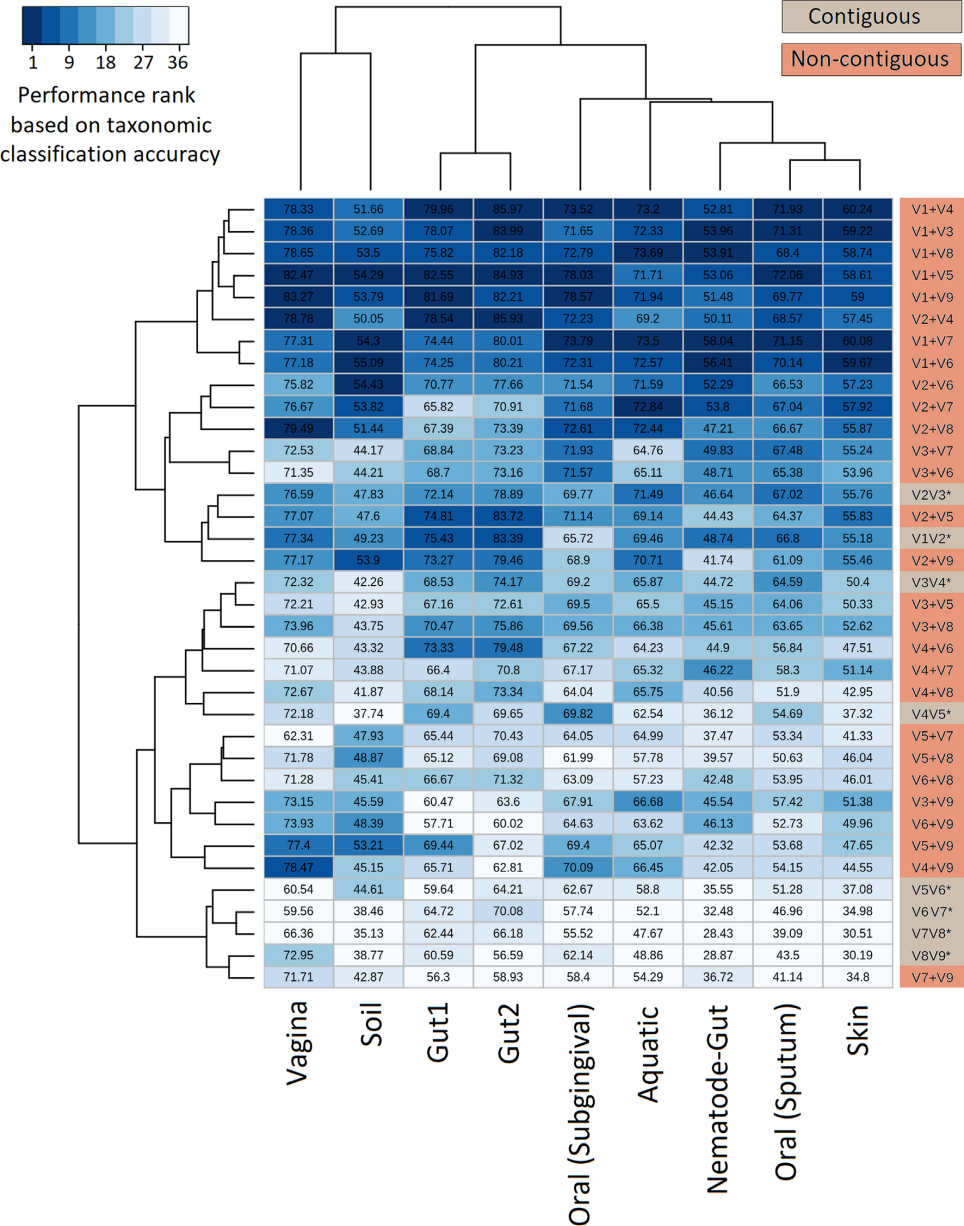
Evaluation of taxonomic classification efficiency on simulated microbiomes. Taxonomic classification efficiency of different combinations of V-regions evaluated on nine simulated microbiome datasets mimicking different environmental niches. Taxonomic classification accuracy in terms of percentages of correct assignments at species level are indicated in the heatmap. The color scale (1–36) depicts the performance rank of different combinations of V-regions (total of 36 combinations) in terms of taxonomic classification accuracy for each of the simulated microbiomes (presented in columns).

The efficiency of the proposed non-contiguous combination of V-regions was further tested on nine additional simulated microbiomes (see Methods) mimicking different environmental and host-associated niches (see Methods, **Supplementary Tables S3**, **S2**, and **S6**). Results pertaining to these simulated microbiomes—namely, gut, skin, vaginal, sub-gingival (oral), sputum (oral), nematode gut, soil, and aquatic are depicted in **Figure 5**. It was interesting to note that optimal classification of reads from the simulated microbiomes pertaining to different niches could be obtained with different combinations of non-contiguous V-regions.

The combination of V1+V4 regions provided the maximum accuracy of classification for skin (60.2%) and one of the gut (86.0%) microbiomes (Gut2), whereas microbiomes pertaining to vaginal and sub-gingival niches were best resolved by the combination V1+V9 (with accuracies of 83.3% and 78.6%, respectively). Optimal classification of sputum microbiome samples (72.1%) could be obtained by another non-contiguous combination, viz. V1+V5 regions, which could also provide relatively more accurate classification for the Gut1 microbiome (82.5%). It was also interesting to note the high variability in classification accuracies of individual V-region combinations while classifying samples pertaining to different environments. For example, while the combination V2+V4 could classify one of the gut microbiomes (Gut2) with 85.93% accuracy, the classification results were not as high when the same combination was used to classify the aquatic microbiome (69.2%). On the other hand, the combination V2+V7 was observed to provide decent classification for the simulated aquatic microbiome (72.8%), while performing not so well for the simulated gut microbiome datasets (65.8% for Gut1 and 70.9% for Gut2). These results further reiterate the need of choosing an optimal combination of V-regions, preferably non-contiguous, for a specific sampled environment.

It may be noted here that the paired-end reads generated for *in silico* evaluation of the utility of different combinations of V-regions were based on HMMs pertaining to the flanking constant regions, as provided by the V-Xtractor program (see Methods). Actual primer design may not always allow generation of reads identical to the *in silico* experiment, and results from a sequencing experiment may slightly vary from the validation results presented. A comparison of the paired-end reads generated in the *in silico* experiments with respect to those which may be obtained by using different sets of primers currently available for 16S rRNA amplicon sequencing is provided in **Supplementary Figure S3**, and **Supplementary Tables S7** and **S8**. **Supplementary Figure S3(A)** and **Supplementary Table S8** additionally depict the specificity of different primer sets that may be used to target various combinations of V-regions with respect to the sequences present in the RDP database. It may be mentioned here that assessment of primer specificity on all sequences from RDP database (a total of 232,163 sequences having length > = 1,200 bp) revealed that the combinations/pairs (either contiguous or non contiguous) involving the V1-region could potentially amplify a lower fraction of sequences compared to other combinations. Apparently, the fraction of sequences that can be amplified by the said combinations is limited by the specificity/universality of the primer for V1-region. The presence of many incomplete/truncated SSU rRNA sequences in RDP database, which might be missing the V1 primer binding sites may also contribute to this observation. The overall results, however, do not indicate any significant deviations in the specificity (fraction of bacterial sequences amplified) of primer pairs targeting non-contiguous V-regions, when compared to the primers targeting contiguously placed V-regions.

It may also be noted that this work did not compare and validate the efficacy of the proposed method in perspective of some recent taxonomic analysis methods which performs exact sequence variant (ESV) analyses (Amir et al., 2017; Callahan et al., 2016, 2). This was primarily because the currently available implementations of such methods expect a significant overlap between the paired-end reads and only work after the two reads are merged (or works with individual reads), thereby making it difficult to make a direct comparison with non-overlapping paired-end reads targeting non-contiguous V-regions. However, one would expect that the combination of V-regions that cannot provide good resolution at genus or species levels will also fail at deeper taxonomic levels like OTUs/sub-OTUs/ESVs, and vice versa.

### Consensus of Multiple Combinations of V-Regions Enables Further Refinement of Taxonomic Profiles

Although better taxonomic classification accuracies can be obtained by using information from multiple V-regions, relatively higher costs and lower throughput serve as deterrents against adoption of long-read sequencing technologies for metagenomic studies. To overcome this bottleneck, we propose a combinatorial strategy (**Figure 1**) that extends the described paired-end sequencing workflow (achievable with a short read sequencing technology like Illumina) for targeting multiple pair-wise combinations of non-contiguous (or contiguous) V-regions (see Methods). The proposed strategy relies on obtaining taxonomic abundance profiles of a microbial community from two paired-end sequencing experiments, each of which targets different pair-wise combinations of V-regions. The two taxonomic profiles are then combined based on (pre-estimated) accuracies of the individual V-regions (targeted in the experiments) in resolving each of the taxonomic groups under consideration.

Considering the fact that human gut is one of the most diverse and densely populated reservoir of microbes, the utility of the combinatorial strategy was assessed with one of the simulated human gut microbiome sample Gut1 (as described earlier). As can be seen from **Figure 5**, the V-region combinations V_1_+V_4_ and V_1_+V_5_ provided highest average classification accuracies for most of the host (human)-associated environmental niches. Consequently, these V-region combinations were targeted for evaluating this combinatorial strategy wherein 5,000 sequence fragments corresponding to each of the V-region combinations (i.e., a total of 10,000 fragments) were sampled from the simulated microbiome. The results obtained with the combinatorial strategy were compared against the results obtained when each of the V-region combinations were targeted separately (with a sequencing depth of 10,000 reads in each case).

Results in **Table 2** indicate that although the V1+V4 and V1+V5 regions can classify the reads with commendable accuracy, the abundance values provided for individual genera deviates from the actual (RDP) lineage by a certain extent. The combinatorial approach was observed to moderate these deviations to a significant extent, and relative abundance of individual genera ascertained by the combinatorial approach exhibited better coherence with the actual lineage. In quantitative terms, while the average deviations (from actual lineage) in relative taxonomic abundance predictions for V1+V4 and V1+V5 combination–based approaches were 17.4% and 11.5%, respectively, the combinatorial approach exhibited a significantly lower average deviation (6.9%) from the actual lineage. Similar improvements were also observed when this approach was tested on microbiomes pertaining to other host-associated/environmental niches (**Supplementary Table S9**). Given that the proposed combinatorial approach does not incur any significant additional sequencing cost and is a simple *in silico* extrapolation of the results obtained with standard pair-end sequencing, adoption of the same would be easy and would enable researchers to explore the taxonomic diversity of different environments with greater accuracy. While certain additional experimental costs for primers, multiplexing barcodes, additional PCR, and handling etc. are expected to be incurred to implement the proposed combinatorial strategy, the actual sequencing (reagents) cost, constituting the bulk of the total expenditure, remains the same. The additional pre-processing and handling efforts can at most be twice compared to the sample handling efforts needed for a single paired-end sequencing experiment. However, the potential benefits in terms of an improved taxonomic resolution are expected to outweigh any inhibitions arising due to the additional, but trivial, pre-processing and handling efforts.

**Table 2:** Utility of proposed combinatorial approach in obtaining refined taxonomic profiles compared to taxonomic abundance estimates obtained with pair-wise combinations of V-regions.

Species	Abundance (%) estimated with full-length 16S reads	Abundance (%) estimated with 10,000 V1+V4 paired-end reads	Abundance (%) estimated with 10,000 V1+V5 paired-end reads	Abundance (%) estimated with combinatorial approach using 5,000 V1+V4 and 5,000 V1+V5 reads
*Faecalibacterium prausnitzii*	11.17	12.24	12.25	11.06
*Bacteroides faecis*	10.69	11.97	11.24	11.36
*Prevotella amnii*	6.73	0.00	6.72	7.28
*Prevotella nigrescens*	6.47	6.98	6.76	6.96
*Megamonas hypermegale*	5.35	6.06	3.53	4.71
*Bacteroides pyogenes*	4.23	4.44	4.33	4.55
*Bacteroides finegoldii*	3.98	4.03	4.13	4.00
*Alistipes putredinis*	3.45	3.73	3.71	3.51
*Roseburia hominis*	2.41	2.70	2.84	2.62
*Bacteroides nordii*	2.18	2.50	2.26	2.16
*Bacteroides eggerthii*	2.15	2.51	2.24	2.15
*Bacteroides helcogenes*	2.09	2.35	2.13	2.11
*Bacteroides caccae*	2.08	2.30	2.32	2.32
*Bacteroides massiliensis*	2.07	2.10	2.13	2.03
*Bacteroides coprocola*	2.04	2.43	2.27	2.21
*Bacteroides salyersiae*	2.04	2.26	2.01	2.12
*Bacteroides stercoris*	2.03	1.92	2.50	2.17
*Bacteroides uniformis*	2.02	2.03	2.04	1.93
*Bacteroides acidifaciens*	2.01	2.30	2.00	2.08
*Proteiniphilum acetatigenes*	2.01	2.21	2.18	2.07
*Bacteroides cellulosilyticus*	1.98	2.16	0.00	1.70
*Bacteroides intestinalis*	1.96	2.02	2.08	2.03
*Roseburia faecis*	1.74	1.94	1.91	1.69
*Roseburia intestinalis*	1.74	2.16	1.91	1.86
*Parasutterella secunda*	1.50	1.74	1.56	1.38
*Roseburia inulinivorans*	1.00	1.00	1.06	1.11
*Phascolarctobacterium succinatutens YIT 12067*	0.99	0.82	0.78	0.80
*Parabacteroides distasonis*	0.90	1.03	1.04	0.74
*Parabacteroides merdae*	0.89	1.07	0.87	0.92
*Parasutterella excrementihominis*	0.82	0.99	0.84	0.75
*Dorea longicatena*	0.78	0.32	0.51	0.32
*Phascolarctobacterium faecium*	0.74	0.81	0.83	0.69
*Blautia producta*	0.70	0.55	0.86	0.61
*Escherichia/Shigella fergusonii*	0.69	0.59	0.00	0.00
*Escherichia/Shigella albertii*	0.57	0.56	0.62	0.71
*Escherichia/Shigella flexneri*	0.56	0.00	0.00	0.00
*Escherichia/Shigella dysenteriae*	0.53	0.50	0.54	0.57
*Dialister invisus*	0.47	0.58	0.50	0.46
*Megasphaera elsdenii*	0.46	0.37	0.47	0.40
*Blautiaglucerasea*	0.45	0.41	0.48	0.61
*Blautia hydrogenotrophica*	0.43	0.44	0.46	0.51
*Blautia schinkii*	0.43	0.47	0.54	0.43
*Mitsuokella jalaludinii*	0.39	0.40	0.42	0.35
*Collinsella aerofaciens*	0.34	0.37	0.42	0.36
*Bifidobacterium longum*	0.32	0.40	0.37	0.38
*Bifidobacterium animalis*	0.32	0.25	0.32	0.29
*Ruminococcus flavefaciens*	0.30	0.21	0.25	0.17
*Blautia hansenii*	0.28	0.33	0.30	0.33
*Megasphaera *sp. *NMBHI-10*	0.28	0.22	0.19	0.17
*Klebsiella pneumoniae*	0.25	0.21	0.29	0.27
**Cumulated percentage deviation from abundance estimated using full-length 16S sequences**	**–**	**17.40**	**11.47**	**6.85**

Results in the table pertain to the simulated human gut microbiome dataset Gut1 (as depicted in **Figure 5**).

## Conclusion

The suggested protocol of targeting non-contiguously placed 16S rRNA V-regions in microbiome studies can yield better taxonomic classification accuracies without any significant additional cost/effort. A simple *in silico* combinatorial strategy further allows building consensus taxonomic profiles from multiple pair-wise combinations of V-regions, while improving accuracy in taxonomic classification. The results of the current study can serve as a guideline for future 16S rRNA amplicon–based microbiome studies and help researchers to choose the most optimal combination of V-regions for their experiment/sampled environment.

## Author Contributions

AD and MH conceived the idea. NP performed the computational analysis with assistance from AD. NP, AD, MH, and SM interpreted the results and drafted the final manuscript.

## Funding

Authors of this study are employees of Tata Consultancy Services Ltd., Pune, India. The company provided support in the form of salaries for authors, but did not have any additional role in the study design, data collection and analysis, decision to publish, or preparation of the manuscript.

## Conflict of Interests Statement

The authors are employees of the Research and Development Division of Tata Consultancy Services Ltd., Pune, India, which is a commercial company. However, this does not alter their adherence to journal policies on sharing data and materials. The authors also declare that no competing interests exist.
