# Supplementary material for: Can Targeting Non-Contiguous V-Regions With Paired-End Sequencing Improve 16S rRNA-Based Taxonomic Resolution of Microbiomes?: An In Silico Evaluation
Source: Front Genet. 2019 Jul 12;10:653. doi: 10.3389/fgene.2019.00653 (PMC6640118; doi:10.3389/fgene.2019.00653)
Supplement: Supplementary file 1 [file Table_1.docx]

**Supplementary Table S1.** Source genera for 16S rRNA sequences included in five 16S gene pools. Randomly drawn sequences from these pools were used in generation of mock datasets.

| **Genus** | **16S gene pool 1** | **16S gene pool 2** | **16S gene pool 3** | **16S gene pool 4** | **16S gene pool 5** |
| --- | --- | --- | --- | --- | --- |
| Acetobacterium | No | No | No | Yes | No |
| Achromobacter | Yes | No | Yes | No | No |
| Acidiphilium | No | No | No | No | Yes |
| Acidithiobacillus | No | Yes | No | No | No |
| Acidovorax | Yes | No | No | No | No |
| Acinetobacter | Yes | No | No | Yes | No |
| Actinobacillus | Yes | No | No | No | No |
| Actinomadura | No | No | Yes | No | Yes |
| Aggregatibacter | No | Yes | No | Yes | Yes |
| Agromyces | No | No | No | No | Yes |
| Alcaligenes | Yes | No | No | No | No |
| Alcanivorax | No | Yes | No | No | No |
| Alicyclobacillus | No | No | No | No | Yes |
| Alkalibacterium | No | No | No | No | Yes |
| Alteromonas | No | No | No | No | Yes |
| Arcobacter | Yes | No | No | No | Yes |
| Arthrobacter | Yes | Yes | Yes | Yes | No |
| Asaia | No | No | Yes | No | No |
| Azoarcus | Yes | No | No | No | No |
| Azospirillum | No | Yes | Yes | No | No |
| Bacillus | No | No | Yes | No | No |
| Bifidobacterium | No | Yes | No | No | Yes |
| Borrelia | No | No | No | Yes | Yes |
| Bosea | No | No | Yes | Yes | No |
| Brachybacterium | No | No | Yes | No | No |
| Bradyrhizobium | No | Yes | Yes | Yes | No |
| Brevibacillus | Yes | No | No | No | No |
| Brevundimonas | Yes | No | No | No | No |
| Brucella | Yes | No | No | No | No |
| Buchnera | Yes | No | Yes | No | Yes |
| Burkholderia | No | No | No | Yes | No |
| Butyrivibrio | Yes | No | Yes | Yes | No |
| Campylobacter | Yes | No | No | No | No |
| Carnobacterium | No | Yes | No | No | No |
| Caulobacter | No | Yes | Yes | No | No |
| Cellulomonas | Yes | No | No | No | Yes |
| Chromobacterium | Yes | No | No | No | Yes |
| Chromohalobacter | No | No | Yes | No | No |
| Chryseobacterium | No | No | No | Yes | No |
| Citrobacter | Yes | No | No | Yes | No |
| Colwellia | No | No | Yes | Yes | No |
| Comamonas | No | No | No | No | Yes |
| Corallococcus | No | Yes | No | Yes | No |
| Corynebacterium | No | No | No | No | Yes |
| Cronobacter | Yes | No | No | Yes | No |
| Curtobacterium | Yes | No | No | Yes | Yes |
| Deinococcus | No | Yes | Yes | No | Yes |
| Delftia | No | No | Yes | No | Yes |
| Desulfosporosinus | Yes | No | No | Yes | No |
| Desulfotomaculum | No | No | Yes | Yes | No |
| Edwardsiella | No | No | Yes | Yes | No |
| Enterococcus | No | Yes | No | No | Yes |
| Erythrobacter | Yes | No | No | No | Yes |
| Eubacterium | No | No | Yes | No | No |
| Exiguobacterium | No | Yes | No | No | No |
| Flavobacterium | Yes | Yes | No | Yes | No |
| Francisella | Yes | Yes | No | No | No |
| Fusobacterium | No | No | No | No | Yes |
| Gallibacterium | No | No | Yes | No | No |
| Geobacillus | No | No | Yes | Yes | No |
| Glaciecola | No | No | No | Yes | Yes |
| Gluconobacter | No | Yes | No | No | No |
| Haemophilus | No | Yes | No | No | No |
| Halobacillus | No | No | No | Yes | No |
| Halomonas | No | Yes | No | Yes | Yes |
| Helicobacter | Yes | No | No | No | No |
| Herbaspirillum | No | No | No | Yes | No |
| Hydrogenophaga | No | No | Yes | No | No |
| Idiomarina | No | No | No | Yes | No |
| Kitasatospora | No | Yes | No | No | No |
| Klebsiella | Yes | No | No | No | No |
| Kocuria | No | No | Yes | No | No |
| Komagataeibacter | No | No | No | No | Yes |
| Lactobacillus | No | No | Yes | No | Yes |
| Lactococcus | Yes | No | No | Yes | No |
| Legionella | No | Yes | No | No | No |
| Leifsonia | No | Yes | No | No | Yes |
| Leptospira | No | Yes | No | No | No |
| Leucobacter | No | Yes | Yes | No | Yes |
| Leuconostoc | Yes | Yes | No | Yes | No |
| Listeria | Yes | No | No | No | No |
| Loktanella | No | Yes | Yes | No | No |
| Lysinibacillus | Yes | No | No | No | No |
| Lysobacter | No | No | No | No | Yes |
| Marinobacter | No | Yes | No | No | Yes |
| Marinobacterium | No | No | Yes | No | No |
| Marinomonas | No | No | No | Yes | Yes |
| Massilia | Yes | No | Yes | Yes | No |
| Methylobacterium | No | No | No | Yes | Yes |
| Microbispora | Yes | No | No | No | No |
| Micromonospora | Yes | Yes | No | No | No |
| Moraxella | No | No | Yes | No | No |
| Moritella | No | No | No | No | Yes |
| Mycoplasma | No | Yes | No | No | No |
| Neisseria | Yes | Yes | No | No | Yes |
| Nitrosomonas | No | No | Yes | No | No |
| Nocardioides | Yes | Yes | No | Yes | No |
| Novosphingobium | Yes | No | Yes | No | No |
| Oceanobacillus | Yes | No | Yes | Yes | No |
| Paenibacillus | Yes | Yes | No | Yes | No |
| Pandoraea | No | No | No | Yes | No |
| Pantoea | No | No | No | Yes | Yes |
| Paracoccus | No | No | No | No | Yes |
| Pectobacterium | No | No | Yes | No | Yes |
| Pediococcus | No | No | No | No | Yes |
| Photobacterium | No | No | Yes | No | Yes |
| Photorhabdus | No | Yes | Yes | No | No |
| Phyllobacterium | No | Yes | No | No | No |
| Planococcus | No | No | No | Yes | No |
| Polaribacter | No | No | Yes | No | No |
| Polynucleobacter | Yes | No | No | No | No |
| Proteus | Yes | No | Yes | No | No |
| Pseudomonas | No | No | No | No | Yes |
| Pseudoxanthomonas | No | Yes | No | No | No |
| Psychrobacter | No | No | Yes | No | No |
| Rahnella | Yes | No | No | No | No |
| Ralstonia | No | No | Yes | Yes | No |
| Rhizobium | No | Yes | Yes | No | Yes |
| Rhodopirellula | No | Yes | Yes | Yes | Yes |
| Rickettsia | No | No | Yes | Yes | No |
| Ruegeria | No | No | No | No | Yes |
| Ruminococcus | No | Yes | No | No | No |
| Salmonella | No | Yes | No | Yes | Yes |
| Selenomonas | No | No | Yes | No | No |
| Serratia | No | Yes | No | Yes | No |
| Shewanella | Yes | No | No | No | Yes |
| Sorangium | Yes | No | Yes | No | No |
| Sphingobium | No | Yes | No | Yes | Yes |
| Spiroplasma | Yes | No | No | No | No |
| Sporolactobacillus | No | No | Yes | Yes | No |
| Staphylococcus | No | Yes | No | Yes | Yes |
| Stenotrophomonas | Yes | Yes | No | Yes | No |
| Streptococcus | No | Yes | No | No | No |
| Streptomyces | No | Yes | Yes | No | No |
| Streptosporangium | No | No | No | No | Yes |
| Taylorella | Yes | Yes | No | No | No |
| Thalassospira | No | Yes | Yes | No | No |
| Thermoanaerobacter | Yes | No | Yes | No | No |
| Thermoanaerobacterium | No | No | No | Yes | No |
| Thermus | No | No | No | Yes | No |
| Thiomonas | Yes | No | Yes | No | No |
| Trueperella | No | Yes | No | Yes | Yes |
| Vibrio | Yes | No | No | No | Yes |
| Virgibacillus | Yes | No | Yes | No | No |
| Weissella | No | Yes | No | No | Yes |
| Xanthomonas | No | Yes | No | Yes | No |
| Xenorhabdus | Yes | No | No | Yes | No |
| Xylella | No | Yes | No | No | No |
