# Supplementary material for: Can Targeting Non-Contiguous V-Regions With Paired-End Sequencing Improve 16S rRNA-Based Taxonomic Resolution of Microbiomes?: An In Silico Evaluation
Source: Front Genet. 2019 Jul 12;10:653. doi: 10.3389/fgene.2019.00653 (PMC6640118; doi:10.3389/fgene.2019.00653)
Supplement: Supplementary file 3 [file Table_3.docx]

**Supplementary Table S3.** Summary of datasets used for deriving taxonomic profiles pertaining to various environmental and host associated niches used to generate simulated microbiomes mimicking the respective environments. Genera level abundances, averaged over all datasets pertaining to an environment, were used to draw respective proportions 16S rRNA genes from the set of downloaded RDP sequences while constructing the simulated microbiome datasets.

| **Sample Identifier** | **Environment** | **No. of Samples** | **Reference** |
| --- | --- | --- | --- |
| Gut1  (Prebiotics) | Gut  (Human) | 283 | Kato *et al.*, 2014  Xiao *et al.*, 2014 |
| Gut2  (HMP) | Gut  (Human) | 306 | Consortium THMP, 2012 |
| Sputum | Oral  Cavity  (Human) | 68 | Cui *et al.*, 2012  Botero *et al.*, 2014 |
| Skin | Skin  (Human) | 149 | Alekseyenko*et al.*, 2013 |
| Sub-gingival | Oral  Cavity  (Human) | 91 | Griffen*et al.*, 2012 |
| Vaginal | Vagina  (Human) | 394 | Romero *et al.*, 2014 |
| Soil | Soil | 18 | DDBJ ID: ERA411828 |
| Nematode | Nematode Gut  *(Litoditis*  *Marina)* | 36 | DDBJ ID: SRP064694 |
| Aquatic | Aquatic | 20 | Muscarella *et al.*, 2019 |
