# Supplementary material for: Can Targeting Non-Contiguous V-Regions With Paired-End Sequencing Improve 16S rRNA-Based Taxonomic Resolution of Microbiomes?: An In Silico Evaluation
Source: Front Genet. 2019 Jul 12;10:653. doi: 10.3389/fgene.2019.00653 (PMC6640118; doi:10.3389/fgene.2019.00653)
Supplement: Supplementary file 8 [file Table_8.docx]

**Supplementary Table S8.** An evaluation of specificity of sets of primers currently available for 16S rRNA amplicon sequencing when used for targeting non-contiguous pairs of V-regions. (Specificity evaluated while allowing a maximum mismatch of 2 base pairs)

| **Target combination of V-regions** | **Set of available primers suitable for targeting the given combination** | **Forward primer sequence**  **(5’-3’)** | **Reverse primer sequence**  **(5’-3’)** | **Number of 16S sequences the primer set is specific to**  **(out of 232,163 full-length sequences from RDP^#^)** | **Percentage (%) of 16S sequences the primer set is specific to**  **(out of 232,163 full-length sequences from RDP^#^)** |
| --- | --- | --- | --- | --- | --- |
| V1+V4 | 68F - 798R | TNANACATGCAAGTCGRRCG | AGGATTAGATACCCT | 130870 | 56.37 |
| V1+V3 | 68F - U529R | TNANACATGCAAGTCGRRCG | GCCAGCMGCCGCGGT | 130814 | 56.35 |
| V1+V8 | 68F - 1401R | TNANACATGCAAGTCGRRCG | GGGTCTTGTACACACCG | 124404 | 53.58 |
| V1+V7 | 68F - GM12R | TNANACATGCAAGTCGRRCG | GAGGAAGGTGKGGATGACG | 130049 | 56.02 |
| V1+V6 | 68F - 1061R | TNANACATGCAAGTCGRRCG | GTCGTCAGCTCGTGYYG | 130902 | 56.38 |
| V1+V5 | 68F - 908R | TNANACATGCAAGTCGRRCG | AACTCAAAKGAATTGACG | 130565 | 56.24 |
| V1+V9 | 68F - 1407R | TNANACATGCAAGTCGRRCG | GYACACACCGCCCGTC | 123605 | 53.24 |
| V2+V4 | pBR-V1.ASF - 798R | AGTGGCGGACGGGTGAGTAA | AGGATTAGATACCCT | 199478 | 85.92 |
| V2+V8 | pBR-V1.ASF - 1401R | AGTGGCGGACGGGTGAGTAA | GGGTCTTGTACACACCG | 186137 | 80.18 |
| V2+V6 | pBR-V1.ASF - 1061R | AGTGGCGGACGGGTGAGTAA | GTCGTCAGCTCGTGYYG | 199237 | 85.82 |
| V2+V7 | pBR-V1.ASF - GM12R | AGTGGCGGACGGGTGAGTAA | GAGGAAGGTGKGGATGACG | 198295 | 85.41 |
| V2V3* | pBR-V1.ASF - U529R | AGTGGCGGACGGGTGAGTAA | GCCAGCMGCCGCGGT | 199433 | 85.90 |
| V2+V9 | pBR-V1.ASF - 1407R | AGTGGCGGACGGGTGAGTAA | GYACACACCGCCCGTC | 184634 | 79.53 |
| V1V2* | 68F - 338R | TNANACATGCAAGTCGRRCG | ACTCCTACGGGAGGCAGCA | 130110 | 56.04 |
| V3+V8 | 347F - 1401R | GGAGGCAGCAGTRRGGAAT | GGGTCTTGTACACACCG | 207895 | 89.55 |
| V3+V7 | 347F - GM12R | GGAGGCAGCAGTRRGGAAT | GAGGAAGGTGKGGATGACG | 225644 | 97.19 |
| V2+V5 | pBR-V1.ASF - 908R | AGTGGCGGACGGGTGAGTAA | AACTCAAAKGAATTGACG | 198718 | 85.59 |
| V3+V6 | 347F - 1061R | GGAGGCAGCAGTRRGGAAT | GTCGTCAGCTCGTGYYG | 226892 | 97.73 |
| V3+V9 | 347F - 1407R | GGAGGCAGCAGTRRGGAAT | GYACACACCGCCCGTC | 205624 | 88.57 |
| V3+V5 | 347F - 908R | GGAGGCAGCAGTRRGGAAT | AACTCAAAKGAATTGACG | 226145 | 97.41 |
| * contiguous combinations  ^#^ see Methods | | | | | |
