# Supplementary material for: Can Targeting Non-Contiguous V-Regions With Paired-End Sequencing Improve 16S rRNA-Based Taxonomic Resolution of Microbiomes?: An In Silico Evaluation
Source: Front Genet. 2019 Jul 12;10:653. doi: 10.3389/fgene.2019.00653 (PMC6640118; doi:10.3389/fgene.2019.00653)
Supplement: Supplementary file 9 [file Table_9.docx]

**Supplementary Table S9A.** Utility of proposed combinatorial approach in obtaining refined taxonomic profiles compared to taxonomic abundance estimates obtained with pair-wise combinations of V-regions. Results in the table pertain to the simulated human gut microbiome dataset **Gut2** (as depicted in Figure 5).

| **Species** | **Abundance (%) estimated with full-length 16S reads** | **Abundance (%) estimated with 10000 V1+V4 paired-end reads** | **Abundance (%) estimated with 10000 V1+V5 paired-end reads** | **Abundance (%) estimated with combinatorial approach using 5000 V1+V4 and 5000 V1+V5 reads** |
| --- | --- | --- | --- | --- |
| *Bacteroides_faecis_(T)* | 13.23 | 13.23 | 13.31 | 13.54 |
| *Alistipes_putredinis_(T)* | 9.14 | 9.24 | 9.85 | 9.54 |
| *Faecalibacterium_prausnitzii_(T)* | 8.42 | 8.67 | 8.64 | 8.18 |
| *Bacteroides_pyogenes_(T)* | 5.24 | 4.99 | 5.51 | 4.94 |
| *Bacteroides_finegoldii_(T)* | 4.89 | 4.93 | 5.33 | 4.72 |
| *Parabacteroides_merdae_(T)* | 3.57 | 3.81 | 3.70 | 3.38 |
| *Parabacteroides_distasonis_(T)* | 3.23 | 3.19 | 3.36 | 3.41 |
| *Oscillibacter_valericigenes_(T)* | 2.95 | 3.23 | 3.01 | 3.03 |
| *Bacteroides_acidifaciens_(T)* | 2.81 | 2.71 | 2.88 | 2.88 |
| *Bacteroides_salyersiae_(T)* | 2.72 | 2.85 | 2.98 | 2.60 |
| *Bacteroides_coprocola_(T)* | 2.65 | 2.29 | 2.82 | 2.79 |
| *Bacteroides_massiliensis_(T)* | 2.64 | 2.69 | 2.94 | 2.79 |
| *Bacteroides_intestinalis_(T)* | 2.63 | 2.60 | 2.77 | 2.35 |
| *Bacteroides_uniformis_(T)* | 2.62 | 2.89 | 2.74 | 2.69 |
| *Bacteroides_stercoris_(T)* | 2.59 | 2.53 | 2.60 | 2.88 |
| *Bacteroides_cellulosilyticus_(T)* | 2.58 | 2.45 | 0.00 | 2.60 |
| *Bacteroides_eggerthii_(T)* | 2.53 | 2.71 | 2.67 | 2.62 |
| *Bacteroides_caccae_(T)* | 2.50 | 2.74 | 2.72 | 2.69 |
| *Proteiniphilum_acetatigenes_(T)* | 2.48 | 2.70 | 2.20 | 2.81 |
| *Bacteroides_helcogenes_(T)* | 2.47 | 2.74 | 2.41 | 2.37 |
| *Bacteroides_nordii_(T)* | 2.41 | 2.68 | 2.51 | 2.46 |
| *Ruminococcus_flavefaciens_(T)* | 1.51 | 1.00 | 1.29 | 1.08 |
| *Ruminococcus_albus_(T)* | 1.45 | 1.24 | 1.30 | 1.26 |
| *Roseburia_hominis_(T)* | 1.08 | 1.11 | 1.10 | 1.12 |
| *Odoribacter_laneus_(T)* | 0.93 | 0.80 | 1.05 | 0.94 |
| *Roseburia_intestinalis_(T)* | 0.88 | 0.81 | 0.95 | 0.78 |
| *Parasutterella_secunda_(T)* | 0.86 | 1.08 | 0.95 | 0.76 |
| *Phascolarctobacterium_succinatutens_YIT_12067* | 0.81 | 0.73 | 0.55 | 0.72 |
| *Roseburia_faecis_(T)* | 0.79 | 0.74 | 0.73 | 0.75 |
| *Dialister_invisus_(T)* | 0.68 | 0.72 | 0.66 | 0.71 |
| *Phascolarctobacterium_faecium_(T)* | 0.60 | 0.63 | 0.62 | 0.54 |
| *Prevotella_amnii_(T)* | 0.55 | 0.00 | 0.74 | 0.58 |
| *Prevotella_nigrescens_(T)* | 0.54 | 0.57 | 0.51 | 0.52 |
| *Roseburia_inulinivorans_(T)* | 0.49 | 0.64 | 0.48 | 0.55 |
| *Flavonifractor_plautii_(T)* | 0.46 | 0.45 | 0.41 | 0.55 |
| *Blautia_producta_(T)* | 0.46 | 0.23 | 0.47 | 0.46 |
| *Coprococcus_catus_(T)* | 0.42 | 0.45 | 0.39 | 0.50 |
| *Parasutterella_excrementihominis_(T)* | 0.40 | 0.41 | 0.36 | 0.37 |
| *Dialister_pneumosintes_(T)* | 0.38 | 0.39 | 0.47 | 0.33 |
| *Dorea_longicatena_(T)* | 0.38 | 0.14 | 0.09 | 0.12 |
| *Ruminococcus_bromii_(T)* | 0.28 | 0.23 | 0.15 | 0.26 |
| *Blautia_hydrogenotrophica_(T)* | 0.25 | 0.24 | 0.27 | 0.27 |
| *Coprococcus_eutactus_(T)* | 0.24 | 0.28 | 0.25 | 0.24 |
| *Blautia_glucerasea_(T)* | 0.22 | 0.24 | 0.22 | 0.22 |
| *Blautia_schinkii_(T)* | 0.21 | 0.22 | 0.25 | 0.16 |
| *Blautia_wexlerae_(T)* | 0.21 | 0.19 | 0.17 | 0.26 |
| *Butyricicoccus_pullicaecorum_(T)* | 0.19 | 0.18 | 0.20 | 0.24 |
| *Butyricimonas_synergistica_(T)* | 0.15 | 0.15 | 0.10 | 0.20 |
| *Blautia_hansenii_(T)* | 0.14 | 0.11 | 0.19 | 0.12 |
| *Ruminococcus_faecis_(T)* | 0.11 | 0.11 | 0.13 | 0.11 |
| **Cumulated Percentage Deviation from abundance estimated using full-length 16S sequences** | - | 6.32 | 8.64 | 5.67 |

**Supplementary Table S9B.** Utility of proposed combinatorial approach in obtaining refined taxonomic profiles compared to taxonomic abundance estimates obtained with pair-wise combinations of V-regions. Results in the table pertain to the simulated human gut microbiome dataset **Sputum** (as depicted in Figure 5).

| **Species** | **Abundance (%) estimated with full-length 16S reads** | **Abundance (%) estimated with 10000 V1+V4 paired-end reads** | **Abundance (%) estimated with 10000 V1+V5 paired-end reads** | **Abundance (%) estimated with combinatorial approach using 5000 V1+V4 and 5000 V1+V5 reads** |
| --- | --- | --- | --- | --- |
| *Prevotella_amnii_(T)* | 8.05 | 0.00 | 9.60 | 8.81 |
| *Prevotella_nigrescens_(T)* | 7.85 | 9.65 | 9.08 | 7.95 |
| *Streptococcus_salivarius_(T)* | 7.64 | 8.48 | 8.91 | 8.24 |
| *Streptococcus_suis_(T)* | 6.19 | 7.29 | 7.15 | 6.59 |
| *Granulicatella_adiacens_(T)* | 5.81 | 6.72 | 3.32 | 5.43 |
| *Fusobacterium_nucleatum_(T)* | 4.59 | 4.59 | 4.82 | 4.34 |
| *Streptococcus_agalactiae_(T)* | 4.22 | 5.14 | 4.83 | 4.73 |
| *Staphylococcus_aureus_(T)* | 4.03 | 4.64 | 4.83 | 4.35 |
| *Streptococcus_pyogenes_(T)* | 3.39 | 3.52 | 0.19 | 3.27 |
| *Neisseria_meningitidis* | 2.84 | 2.72 | 1.58 | 2.05 |
| *Streptococcus_gallolyticus_(T)* | 2.31 | 2.65 | 2.62 | 2.66 |
| *Rothia_dentocariosa_(T)* | 2.20 | 2.49 | 2.67 | 2.33 |
| *Streptococcus_dysgalactiae_(T)* | 2.04 | 2.40 | 2.42 | 2.35 |
| *Veillonella_parvula_(T)* | 2.04 | 1.98 | 2.24 | 2.23 |
| *Propionibacterium_acnes_(T)* | 1.92 | 2.07 | 2.12 | 2.41 |
| *Rothia_aeria_(T)* | 1.90 | 2.44 | 2.45 | 2.19 |
| *Veillonella_tobetsuensis_(T)* | 1.90 | 2.12 | 2.35 | 1.92 |
| *Streptococcus_pneumoniae_(T)* | 1.74 | 0.72 | 0.88 | 0.89 |
| *Rothia_mucilaginosa_(T)* | 1.65 | 1.95 | 1.20 | 1.47 |
| *Haemophilus_aegyptius_(T)* | 1.58 | 1.91 | 1.61 | 1.61 |
| *Gemella_sanguinis_(T)* | 1.50 | 1.59 | 1.61 | 1.61 |
| *Gemella_haemolysans_(T)* | 1.43 | 1.73 | 1.55 | 1.37 |
| *Actinomyces_neuii_(T)* | 1.39 | 1.60 | 1.86 | 1.68 |
| *Gemella_bergeri_(T)* | 1.38 | 1.32 | 1.53 | 1.76 |
| *Gemella_morbillorum_(T)* | 1.37 | 1.09 | 1.23 | 1.22 |
| *Dolosigranulum_pigrum_(T)* | 1.36 | 1.51 | 1.52 | 1.71 |
| *Veillonella_criceti_(T)* | 1.35 | 0.79 | 0.68 | 0.68 |
| *Streptococcus_equi_(T)* | 1.34 | 1.71 | 1.64 | 1.29 |
| *Streptococcus_infantarius_(T)* | 1.15 | 1.31 | 1.28 | 1.08 |
| *Pelomonas_saccharophila_(T)* | 1.10 | 0.28 | 0.11 | 0.38 |
| *Rothia_endophytica* | 0.93 | 0.93 | 0.96 | 0.99 |
| *Staphylococcus_warneri_(T)* | 0.91 | 1.12 | 0.90 | 0.93 |
| *Acinetobacter_baumannii_(T)* | 0.85 | 0.93 | 0.90 | 0.94 |
| *Veillonella_atypica_(T)* | 0.77 | 0.00 | 0.00 | 0.00 |
| *Rothia_amarae_(T)* | 0.71 | 1.01 | 0.90 | 0.62 |
| *Veillonella_denticariosi_(T)* | 0.71 | 1.01 | 0.36 | 0.57 |
| *Veillonella_ratti_(T)* | 0.69 | 0.36 | 0.00 | 0.11 |
| *Anoxybacillus_rupiensis_(T)* | 0.68 | 0.87 | 0.85 | 0.74 |
| *Actinomyces_coleocanis_(T)* | 0.68 | 0.70 | 0.82 | 0.77 |
| *Staphylococcus_cohnii_(T)* | 0.60 | 0.67 | 0.57 | 0.50 |
| *Streptococcus_constellatus_(T)* | 0.59 | 0.83 | 0.66 | 0.60 |
| *Peptostreptococcus_russellii_(T)* | 0.55 | 0.59 | 0.76 | 0.62 |
| *Solobacterium_moorei_(T)* | 0.54 | 0.65 | 0.68 | 0.57 |
| *Staphylococcus_hominis_(T)* | 0.54 | 0.59 | 0.63 | 0.54 |
| *Parvimonas_micra_(T)* | 0.54 | 0.65 | 0.73 | 0.54 |
| *Stenotrophomonas_rhizophila_(T)* | 0.53 | 0.53 | 0.46 | 0.58 |
| *Streptococcus_iniae_(T)* | 0.50 | 0.62 | 0.54 | 0.39 |
| *Gemella_palaticanis_(T)* | 0.49 | 0.59 | 0.43 | 0.66 |
| *Peptostreptococcus_anaerobius_(T)* | 0.46 | 0.54 | 0.65 | 0.52 |
| *Streptococcus_pseudoporcinus_(T)* | 0.46 | 0.37 | 0.32 | 0.20 |
| **Cumulated Percentage Deviation from abundance estimated using full-length 16S sequences** | - | 24.30 | 24.35 | 12.64 |

**Supplementary Table S9C.** Utility of proposed combinatorial approach in obtaining refined taxonomic profiles compared to taxonomic abundance estimates obtained with pair-wise combinations of V-regions. Results in the table pertain to the simulated human gut microbiome dataset **Sub-gingival** (as depicted in Figure 5).

| **Species** | **Abundance (%) estimated with full-length 16S reads** | **Abundance (%) estimated with 10000 V1+V4 paired-end reads** | **Abundance (%) estimated with 10000 V1+V5 paired-end reads** | **Abundance (%) estimated with combinatorial approach using 5000 V1+V4 and 5000 V1+V5 reads** |
| --- | --- | --- | --- | --- |
| *Fusobacterium_nucleatum_(T)* | 19.16 | 21.85 | 19.74 | 19.69 |
| *Prevotella_nigrescens_(T)* | 11.17 | 12.88 | 12.66 | 12.57 |
| *Prevotella_amnii_(T)* | 10.27 | 0.00 | 11.42 | 10.43 |
| *Parvimonas_micra_(T)* | 4.41 | 5.50 | 5.13 | 4.83 |
| *Streptococcus_salivarius_(T)* | 4.40 | 5.36 | 5.07 | 4.52 |
| *Streptococcus_suis_(T)* | 3.63 | 4.47 | 3.80 | 4.26 |
| *Streptococcus_agalactiae_(T)* | 2.85 | 3.32 | 3.28 | 3.13 |
| *Streptococcus_pyogenes_(T)* | 2.19 | 2.68 | 0.16 | 2.40 |
| *Capnocytophaga_canimorsus_(T)* | 2.12 | 2.61 | 2.13 | 1.91 |
| *Granulicatella_adiacens_(T)* | 1.85 | 2.00 | 1.03 | 1.59 |
| *Porphyromonas_crevioricanis_(T)* | 1.84 | 2.36 | 1.99 | 1.84 |
| *Campylobacter_lari_(T)* | 1.77 | 0.42 | 0.42 | 0.60 |
| *Treponema_maltophilum_(T)* | 1.57 | 1.15 | 1.62 | 1.31 |
| *Acinetobacter_baumannii_(T)* | 1.45 | 1.66 | 1.39 | 1.37 |
| *Streptococcus_gallolyticus_(T)* | 1.45 | 1.87 | 1.57 | 1.81 |
| *Fusobacterium_necrophorum_(T)* | 1.38 | 1.68 | 1.65 | 1.56 |
| *Streptococcus_dysgalactiae_(T)* | 1.37 | 1.74 | 1.69 | 1.35 |
| *Neisseria_meningitidis* | 1.31 | 1.50 | 0.73 | 0.96 |
| *Leptotrichia_buccalis_(T)* | 1.25 | 1.53 | 1.23 | 1.12 |
| *Porphyromonas_somerae_(T)* | 1.25 | 1.48 | 1.47 | 1.19 |
| *Enhydrobacter_aerosaccus_(T)* | 1.18 | 1.37 | 1.17 | 1.43 |
| *Aggregatibacter_aphrophilus_(T)* | 1.16 | 1.10 | 1.20 | 1.19 |
| *Actinomyces_neuii_(T)* | 1.13 | 1.37 | 1.23 | 1.29 |
| *Filifactor_villosus_(T)* | 1.11 | 1.10 | 1.29 | 1.17 |
| *Fusobacterium_varium_(T)* | 1.07 | 1.21 | 1.27 | 1.19 |
| *Reyranella_massiliensis* | 1.07 | 1.34 | 0.97 | 1.14 |
| *Streptococcus_pneumoniae_(T)* | 1.07 | 0.63 | 0.54 | 0.53 |
| *Treponema_lecithinolyticum_(T)* | 0.97 | 1.23 | 1.00 | 1.03 |
| *Veillonella_parvula_(T)* | 0.94 | 0.89 | 0.94 | 0.85 |
| *Streptococcus_equi_(T)* | 0.90 | 0.95 | 0.97 | 0.79 |
| *Treponema_amylovorum_(T)* | 0.89 | 1.11 | 1.00 | 0.78 |
| *Veillonella_tobetsuensis_(T)* | 0.82 | 0.97 | 0.93 | 0.95 |
| *Fusobacterium_mortiferum_(T)* | 0.75 | 0.45 | 0.27 | 0.24 |
| *Treponema_socranskii_(T)* | 0.71 | 0.97 | 0.81 | 0.82 |
| *Streptococcus_infantarius_(T)* | 0.70 | 0.69 | 0.57 | 0.72 |
| *Porphyromonas_asaccharolytica_DSM_20707* | 0.69 | 0.87 | 0.76 | 0.75 |
| *Leptotrichia_wadei_(T)* | 0.68 | 0.90 | 0.73 | 0.65 |
| *Porphyromonas_endodontalis_(T)* | 0.65 | 0.00 | 0.00 | 0.00 |
| *Acinetobacter_calcoaceticus_(T)* | 0.64 | 0.65 | 0.45 | 0.69 |
| *Leptotrichia_goodfellowii_(T)* | 0.64 | 0.86 | 0.61 | 0.72 |
| *Veillonella_criceti_(T)* | 0.63 | 0.37 | 0.25 | 0.28 |
| *Actinomyces_coleocanis_(T)* | 0.60 | 0.69 | 0.76 | 0.60 |
| *Porphyromonas_gulae_(T)* | 0.60 | 0.00 | 0.00 | 0.00 |
| *Sphingobacterium_spiritivorum_(T)* | 0.57 | 0.40 | 0.52 | 0.57 |
| *Catonella_morbi_(T)* | 0.57 | 0.79 | 0.64 | 0.50 |
| *Porphyromonas_cansulci_(T)* | 0.54 | 0.60 | 0.67 | 0.53 |
| *Rothia_aeria_(T)* | 0.53 | 0.66 | 0.63 | 0.56 |
| *Leptotrichia_hofstadii_(T)* | 0.51 | 0.56 | 0.72 | 0.66 |
| *Acinetobacter_lwoffii_(T)* | 0.49 | 0.52 | 0.40 | 0.40 |
| *Capnocytophaga_cynodegmi_(T)* | 0.49 | 0.66 | 0.49 | 0.55 |
| **Cumulated Percentage Deviation from abundance estimated using full-length 16S sequences** | - | 29.18 | 16.17 | 11.42 |

**Supplementary Table S9D.** Utility of proposed combinatorial approach in obtaining refined taxonomic profiles compared to taxonomic abundance estimates obtained with pair-wise combinations of V-regions. Results in the table pertain to the simulated human gut microbiome dataset **Skin** (as depicted in Figure 5).

| **Species** | **Abundance (%) estimated with full-length 16S reads** | **Abundance (%) estimated with 10000 V1+V4 paired-end reads** | **Abundance (%) estimated with 10000 V1+V5 paired-end reads** | **Abundance (%) estimated with combinatorial approach using 5000 V1+V4 and 5000 V1+V5 reads** |
| --- | --- | --- | --- | --- |
| *Staphylococcus_aureus_(T)* | 20.21 | 22.76 | 24.06 | 22.83 |
| *Propionibacterium_acnes_(T)* | 11.28 | 13.11 | 13.41 | 12.33 |
| *Staphylococcus_warneri_(T)* | 3.81 | 4.26 | 4.43 | 4.44 |
| *Methylobacterium_populi_(T)* | 3.28 | 0.41 | 0.55 | 0.55 |
| *Cupriavidus_taiwanensis_(T)* | 2.96 | 2.70 | 2.56 | 2.66 |
| *Schlegelella_thermodepolymerans_(T)* | 2.92 | 3.89 | 3.68 | 3.44 |
| *Staphylococcus_cohnii_(T)* | 2.55 | 2.74 | 3.13 | 2.64 |
| *Staphylococcus_hominis_(T)* | 2.45 | 2.83 | 2.80 | 2.91 |
| *Cupriavidus_basilensis_(T)* | 2.40 | 2.70 | 2.87 | 2.56 |
| *Uruburuella_suis_(T)* | 2.39 | 2.65 | 2.84 | 2.88 |
| *Corynebacterium_diphtheriae_(T)* | 2.35 | 2.91 | 2.76 | 2.35 |
| *Corynebacterium_glutamicum_(T)* | 2.27 | 2.61 | 2.98 | 3.09 |
| *Cupriavidus_respiraculi_(T)* | 2.06 | 2.35 | 1.85 | 2.00 |
| *Staphylococcus_sciuri_(T)* | 1.88 | 2.22 | 2.09 | 1.84 |
| *Micrococcus_yunnanensis_(T)* | 1.55 | 0.00 | 0.00 | 0.00 |
| *Methylobacterium_komagatae_(T)* | 1.47 | 1.41 | 1.12 | 1.14 |
| *Streptococcus_salivarius_(T)* | 1.47 | 1.50 | 1.52 | 1.44 |
| *Methylobacterium_goesingense_(T)* | 1.45 | 1.74 | 1.57 | 1.70 |
| *Dermacoccus_nishinomiyaensis_(T)* | 1.38 | 1.89 | 1.54 | 1.76 |
| *Corynebacterium_bovis_(T)* | 1.32 | 1.50 | 1.65 | 1.53 |
| *Staphylococcus_equorum_(T)* | 1.28 | 1.65 | 1.46 | 1.43 |
| *Streptococcus_suis_(T)* | 1.26 | 1.33 | 1.59 | 1.57 |
| *Methylobacterium_hispanicum_(T)* | 1.23 | 0.00 | 0.00 | 0.00 |
| *Finegoldia_magna_(T)* | 1.20 | 1.30 | 1.37 | 1.43 |
| *Schlegelella_aquatica_(T)* | 1.20 | 0.00 | 1.48 | 1.92 |
| *Ralstonia_syzygii* | 1.18 | 0.26 | 0.33 | 0.43 |
| *Cupriavidus_pauculus_(T)* | 1.13 | 1.41 | 1.30 | 1.27 |
| *Staphylococcus_capitis_(T)* | 1.13 | 1.28 | 1.28 | 1.31 |
| *Geobacillus_stearothermophilus_(T)* | 1.05 | 1.00 | 0.84 | 1.05 |
| *Wautersia_numazuensis_(T)* | 1.04 | 0.13 | 0.09 | 0.15 |
| *Methylobacterium_mesophilicum_(T)* | 0.99 | 1.09 | 0.88 | 0.78 |
| *Staphylococcus_pasteuri_(T)* | 0.95 | 1.09 | 1.10 | 1.45 |
| *Cupriavidus_campinensis_(T)* | 0.93 | 1.04 | 0.95 | 1.18 |
| *Staphylococcus_carnosus_(T)* | 0.93 | 0.96 | 0.66 | 0.91 |
| *Cupriavidus_alkaliphilus_(T)* | 0.91 | 1.06 | 0.13 | 0.84 |
| *Methylobacterium_rhodesianum_(T)* | 0.90 | 1.06 | 1.17 | 1.05 |
| *Methylobacterium_marchantiae_(T)* | 0.87 | 0.59 | 0.71 | 0.69 |
| *Lactobacillus_plantarum_(T)* | 0.86 | 1.15 | 0.90 | 1.33 |
| *Corynebacterium_ulcerans_(T)* | 0.84 | 0.33 | 0.62 | 0.34 |
| *Propionibacterium_acidipropionici_(T)* | 0.84 | 0.00 | 0.00 | 0.00 |
| *Propionibacterium_acidifaciens_(T)* | 0.82 | 0.91 | 0.95 | 0.92 |
| *Staphylococcus_succinus_(T)* | 0.82 | 0.91 | 0.95 | 1.01 |
| *Propionibacterium_freudenreichii_(T)* | 0.82 | 1.06 | 0.93 | 0.99 |
| *Geobacillus_thermodenitrificans_(T)* | 0.80 | 0.63 | 0.60 | 0.53 |
| *Cupriavidus_sp._ASC-64* | 0.79 | 0.63 | 0.57 | 0.65 |
| *Geobacillus_thermoleovorans_(T)* | 0.78 | 0.57 | 0.02 | 0.39 |
| *Methylobacterium_brachiatum_(T)* | 0.77 | 0.00 | 0.00 | 0.00 |
| *Stenotrophomonas_rhizophila_(T)* | 0.77 | 0.98 | 0.71 | 0.82 |
| *Reyranella_massiliensis* | 0.76 | 0.70 | 0.90 | 0.73 |
| *Streptococcus_pyogenes_(T)* | 0.74 | 0.72 | 0.11 | 0.75 |
| **Cumulated Percentage Deviation from abundance estimated using full-length 16S sequences** | - | 24.15 | 26.99 | 22.65 |

**Supplementary Table S9E.** Utility of proposed combinatorial approach in obtaining refined taxonomic profiles compared to taxonomic abundance estimates obtained with pair-wise combinations of V-regions. Results in the table pertain to the simulated **Soil** microbiome dataset (as depicted in Figure 5).

| **Species** | **Abundance (%) estimated with full-length 16S reads** | **Abundance (%) estimated with 10000 V1+V4 paired-end reads** | **Abundance (%) estimated with 10000 V1+V5 paired-end reads** | **Abundance (%) estimated with combinatorial approach using 5000 V1+V4 and 5000 V1+V5 reads** |
| --- | --- | --- | --- | --- |
| *Bradyrhizobium_pachyrhizi_(T)* | 18.72 | 16.90 | 14.83 | 14.67 |
| *Rhodomicrobium_vannielii* | 5.92 | 4.24 | 7.93 | 6.47 |
| *Gemmata_obscuriglobus_(T)* | 5.23 | 7.60 | 7.11 | 6.93 |
| *Gemmatimonas_aurantiaca_(T)* | 5.16 | 7.89 | 6.41 | 6.78 |
| *Ktedonobacter_racemifer_(T)* | 5.03 | 7.87 | 6.94 | 7.11 |
| *Bradyrhizobium_diazoefficiens_USDA_110* | 4.29 | 0.00 | 2.59 | 3.15 |
| *Bradyrhizobium_japonicum_(T)* | 3.97 | 0.00 | 0.00 | 0.00 |
| *Bradyrhizobium_liaoningense_(T)* | 3.90 | 0.00 | 0.00 | 0.00 |
| *Aquisphaera_giovannonii_(T)* | 3.80 | 5.34 | 4.85 | 5.62 |
| *Gaiella_occulta_(T)* | 2.23 | 3.19 | 2.99 | 2.52 |
| *Mycobacterium_leprae* | 2.17 | 3.14 | 2.42 | 2.72 |
| *Bradyrhizobium_canariense_(T)* | 2.16 | 3.19 | 4.89 | 3.99 |
| *Phenylobacterium_muchangponense* | 2.09 | 1.54 | 1.56 | 1.72 |
| *Bradyrhizobium_sp._OO99* | 1.93 | 0.00 | 0.00 | 0.00 |
| *Bradyrhizobium_rifense* | 1.61 | 0.00 | 0.00 | 0.00 |
| *Burkholderia_fungorum_(T)* | 1.52 | 1.16 | 1.92 | 1.82 |
| *Phenylobacterium_composti_(T)* | 1.45 | 2.44 | 1.88 | 2.24 |
| *Bradyrhizobium_sp._LMTR_21* | 1.44 | 1.91 | 1.75 | 1.86 |
| *Pedomicrobium_ferrugineum_(T)* | 1.42 | 2.11 | 1.94 | 1.79 |
| *Pedomicrobium_australicum_(T)* | 1.33 | 2.24 | 0.00 | 1.75 |
| *Pedomicrobium_manganicum_(T)* | 1.33 | 2.24 | 1.79 | 1.47 |
| *Massilia_aurea_(T)* | 1.29 | 1.96 | 1.88 | 1.70 |
| *Thermoleophilum_album_(T)* | 1.23 | 1.98 | 1.64 | 1.75 |
| *Domibacillus_robiginosus_(T)* | 1.15 | 1.87 | 1.88 | 1.80 |
| *Acidisoma_tundrae_(T)* | 1.11 | 1.67 | 1.27 | 1.70 |
| *Domibacillus_sp._NIO-1016* | 1.07 | 1.80 | 1.52 | 1.40 |
| *Acidisoma_sibiricum_(T)* | 0.98 | 1.45 | 1.26 | 1.15 |
| *Dyella_japonica_(T)* | 0.98 | 0.40 | 0.32 | 0.34 |
| *Opitutus_terrae_(T)* | 0.93 | 1.05 | 1.03 | 1.60 |
| *Bradyrhizobium_iriomotense_(T)* | 0.88 | 0.66 | 1.10 | 0.82 |
| *Tumebacillus_ginsengisoli_(T)* | 0.88 | 0.92 | 1.29 | 1.19 |
| *Burkholderia_phenoliruptrix_(T)* | 0.86 | 1.56 | 1.20 | 0.94 |
| *Burkholderia_unamae_(T)* | 0.85 | 0.57 | 0.68 | 0.63 |
| *Burkholderia_phytofirmans_(T)* | 0.84 | 0.62 | 0.67 | 0.54 |
| *Pedomicrobium_americanum_(T)* | 0.82 | 1.14 | 2.80 | 2.06 |
| *Burkholderia_bannensis* | 0.76 | 0.02 | 0.04 | 0.02 |
| *Bradyrhizobium_denitrificans_(T)* | 0.73 | 0.70 | 0.91 | 0.84 |
| *Rhodopila_globiformis_(T)* | 0.72 | 1.19 | 0.95 | 0.97 |
| *Sinomonas_atrocyanea_(T)* | 0.71 | 0.22 | 0.29 | 0.24 |
| *Burkholderia_tuberum_(T)* | 0.66 | 0.83 | 0.67 | 0.82 |
| *Burkholderia_mimosarum_(T)* | 0.66 | 0.44 | 0.38 | 0.39 |
| *Microvirga_sp._BR3299* | 0.66 | 1.03 | 0.80 | 0.90 |
| *Vampirovibrio_chlorellavorus_(T)* | 0.61 | 0.94 | 0.95 | 0.82 |
| *Burkholderia_sediminicola_(T)* | 0.61 | 0.00 | 0.38 | 0.26 |
| *Legionella_pneumophila_(T)* | 0.58 | 0.83 | 0.87 | 0.79 |
| *Burkholderia_udeis* | 0.57 | 0.09 | 0.06 | 0.10 |
| *Chromobacterium_vaccinii_(T)* | 0.54 | 0.73 | 0.82 | 0.66 |
| *Segetibacter_koreensis_(T)* | 0.54 | 0.70 | 0.70 | 0.98 |
| *Phenylobacterium_falsum_(T)* | 0.53 | 0.79 | 0.76 | 0.74 |
| *Phenylobacterium_immobile_(T)* | 0.53 | 0.83 | 1.08 | 1.23 |
| **Cumulated Percentage Deviation from abundance estimated using full-length 16S sequences** | - | 47.96 | 44.05 | 40.99 |

**Supplementary Table S9F.** Utility of proposed combinatorial approach in obtaining refined taxonomic profiles compared to taxonomic abundance estimates obtained with pair-wise combinations of V-regions. Results in the table pertain to the simulated **Aquatic** microbiome dataset (as depicted in Figure 5).

| **Species** | **Abundance (%) estimated with full-length 16S reads** | **Abundance (%) estimated with 10000 V1+V4 paired-end reads** | **Abundance (%) estimated with 10000 V1+V5 paired-end reads** | **Abundance (%) estimated with combinatorial approach using 5000 V1+V4 and 5000 V1+V5 reads** |
| --- | --- | --- | --- | --- |
| *Polynucleobacter_necessarius_(T)* | 31.17 | 36.21 | 36.30 | 36.01 |
| *Polynucleobacter_cosmopolitanus_(T)* | 12.68 | 14.48 | 13.55 | 13.85 |
| *Mycobacterium_leprae* | 5.91 | 6.74 | 6.67 | 6.84 |
| *Luteolibacter_algae_(T)* | 3.90 | 4.73 | 4.64 | 4.50 |
| *Rhodoferax_saidenbachensis_ED16* | 3.13 | 2.00 | 0.82 | 1.78 |
| *Polynucleobacter_acidiphobus_(T)* | 2.60 | 3.04 | 2.95 | 2.83 |
| *Acidovorax_delafieldii_(T)* | 2.35 | 0.58 | 0.37 | 0.46 |
| *Rhodoferax_antarcticus_(T)* | 2.09 | 2.36 | 2.26 | 2.29 |
| *Acidovorax_temperans_(T)* | 1.85 | 0.58 | 0.66 | 0.54 |
| *Methylophilus_methylotrophus_(T)* | 1.69 | 0.86 | 0.59 | 0.80 |
| *Rhodoferax_fermentans_(T)* | 1.69 | 1.87 | 1.85 | 2.17 |
| *Opitutus_terrae_(T)* | 1.60 | 1.85 | 1.91 | 2.03 |
| *Luteolibacter_pohnpeiensis_(T)* | 1.56 | 1.87 | 1.85 | 1.86 |
| *Haliscomenobacter_hydrossis_(T)* | 1.35 | 1.59 | 1.63 | 1.54 |
| *Acidovorax_cattleyae_(T)* | 1.27 | 1.50 | 2.51 | 1.82 |
| *Mycobacterium_iranicum_(T)* | 1.26 | 1.48 | 1.44 | 1.45 |
| *Mycobacterium_novocastrense_(T)* | 1.16 | 1.27 | 1.40 | 1.35 |
| *Mycobacterium_marinum_(T)* | 1.13 | 0.00 | 0.00 | 0.00 |
| *Methylomonas_methanica_(T)* | 1.03 | 0.06 | 0.04 | 0.09 |
| *Mycobacterium_tuberculosis_(T)* | 1.01 | 0.00 | 0.00 | 0.00 |
| *Microbacterium_paraoxydans_(T)* | 0.99 | 1.04 | 1.10 | 0.83 |
| *Algoriphagus_namhaensis* | 0.97 | 1.13 | 1.00 | 1.10 |
| *Polynucleobacter_rarus_(T)* | 0.96 | 1.08 | 1.09 | 0.95 |
| *Mycobacterium_cookii_(T)* | 0.95 | 1.20 | 1.07 | 1.11 |
| *Acidovorax_caeni_(T)* | 0.93 | 1.13 | 1.03 | 1.08 |
| *Mycobacterium_arupense_(T)* | 0.93 | 0.00 | 0.00 | 0.00 |
| *Flavobacterium_degerlachei_(T)* | 0.80 | 0.33 | 0.72 | 0.59 |
| *Methylomonas_koyamae_(T)* | 0.78 | 0.59 | 0.94 | 0.95 |
| *Acidovorax_avenae_(T)* | 0.72 | 0.00 | 0.00 | 0.00 |
| *Methylophilus_leisingeri_(T)* | 0.71 | 0.82 | 1.41 | 0.95 |
| *Rhodomicrobium_vannielii* | 0.71 | 0.39 | 0.88 | 0.71 |
| *Fluviicola_taffensis* | 0.70 | 0.87 | 0.84 | 0.89 |
| *Comamonas_testosteroni_(T)* | 0.67 | 0.65 | 0.68 | 0.76 |
| *Beijerinckia_indica_(T)* | 0.64 | 0.77 | 0.76 | 0.81 |
| *Algoriphagus_antarcticus_(T)* | 0.62 | 0.73 | 0.69 | 0.72 |
| *Acidovorax_radicis_(T)* | 0.61 | 0.00 | 0.00 | 0.00 |
| *Methylocystis_rosea_(T)* | 0.57 | 0.58 | 1.07 | 0.75 |
| *Methylomonas_scandinavica_(T)* | 0.57 | 0.34 | 0.66 | 0.47 |
| *Methylophilus_flavus_(T)* | 0.57 | 0.36 | 0.35 | 0.37 |
| *Stenotrophomonas_rhizophila_(T)* | 0.55 | 0.61 | 0.50 | 0.53 |
| *Methylocystis_hirsuta_(T)* | 0.53 | 0.47 | 0.00 | 0.27 |
| *Comamonas_jiangduensis_(T)* | 0.51 | 0.06 | 0.06 | 0.09 |
| *Algoriphagus_halophilus_(T)* | 0.50 | 0.61 | 0.60 | 0.61 |
| *Algoriphagus_lutimaris_(T)* | 0.49 | 0.52 | 0.60 | 0.45 |
| *Verrucomicrobium_spinosum_(T)* | 0.45 | 0.52 | 0.54 | 0.60 |
| *Acidovorax_konjaci_(T)* | 0.44 | 0.13 | 0.10 | 0.18 |
| *Aquisphaera_giovannonii_(T)* | 0.44 | 0.55 | 0.56 | 0.60 |
| *Belnapia_moabensis_(T)* | 0.44 | 0.52 | 0.50 | 0.54 |
| *Caulobacter_henricii_(T)* | 0.40 | 0.47 | 0.34 | 0.43 |
| *Prosthecobacter_vanneervenii_(T)* | 0.40 | 0.49 | 0.44 | 0.44 |
| **Cumulated Percentage Deviation from abundance estimated using full-length 16S sequences** | - | 25.26 | 27.39 | 24.93 |

**Supplementary Table S9G.** Utility of proposed combinatorial approach in obtaining refined taxonomic profiles compared to taxonomic abundance estimates obtained with pair-wise combinations of V-regions. Results in the table pertain to the simulated human gut microbiome dataset **Vagina** (as depicted in Figure 5). While the chosen pairs of V-regions appear to provide sub-optimal performance for the Vaginal microbiome dataset, using a different set of V-region pairs (e.g. V1+V5 and V1+V7) improves the results of the combinatorial approach.

| **Species** | **Abundance (%) estimated with full-length 16S reads** | **Abundance (%) estimated with 10000 V1+V4 paired-end reads** | **Abundance (%) estimated with 10000 V1+V5 paired-end reads** | **Abundance (%) estimated with combinatorial approach using 5000 V1+V4 and 5000 V1+V5 reads** |
| --- | --- | --- | --- | --- |
| *Lactobacillus_plantarum_(T)* | 24.11 | 26.50 | 24.97 | 24.72 |
| *Lactobacillus_paracasei_(T)* | 14.14 | 15.43 | 14.90 | 14.73 |
| *Lactobacillus_fermentum* | 8.94 | 9.45 | 9.22 | 9.75 |
| *Lactobacillus_delbrueckii_(T)* | 7.71 | 8.43 | 7.83 | 7.95 |
| *Prevotella_amnii_(T)* | 4.99 | 0.00 | 5.34 | 5.30 |
| *Prevotella_nigrescens_(T)* | 4.82 | 5.37 | 5.12 | 5.18 |
| *Sneathia_sanguinegens_(T)* | 4.39 | 4.80 | 4.61 | 4.43 |
| *Atopobium_rimae_(T)* | 2.70 | 1.06 | 0.93 | 0.96 |
| *Lactobacillus_reuteri_(T)* | 2.26 | 2.54 | 2.39 | 2.33 |
| *Lactobacillus_diolivorans_(T)* | 2.10 | 2.29 | 2.45 | 2.34 |
| *Lactobacillus_farraginis_(T)* | 1.60 | 1.63 | 1.38 | 1.38 |
| *Lactobacillus_sakei_(T)* | 1.59 | 1.81 | 1.54 | 1.51 |
| *Lactobacillus_amylovorus_(T)* | 1.22 | 1.40 | 1.29 | 1.43 |
| *Lactobacillus_kimchii_(T)* | 1.13 | 0.37 | 0.42 | 0.29 |
| *Lactobacillus_gasseri_(T)* | 1.04 | 1.18 | 0.99 | 1.03 |
| *Atopobium_minutum_(T)* | 0.98 | 1.08 | 1.04 | 1.11 |
| *Lactobacillus_kefiri_(T)* | 0.97 | 1.17 | 0.97 | 0.94 |
| *Lactobacillus_futsaii* | 0.91 | 0.89 | 0.91 | 0.84 |
| *Lactobacillus_kefiranofaciens_(T)* | 0.86 | 0.99 | 0.89 | 0.80 |
| *Lactobacillus_farciminis* | 0.79 | 0.18 | 0.18 | 0.18 |
| *Finegoldia_magna_(T)* | 0.76 | 0.80 | 0.85 | 0.75 |
| *Lactobacillus_buchneri_(T)* | 0.73 | 0.90 | 0.78 | 0.80 |
| *Parvimonas_micra_(T)* | 0.73 | 0.90 | 0.87 | 0.75 |
| *Lactobacillus_mucosae_(T)* | 0.64 | 0.68 | 0.73 | 0.69 |
| *Lactobacillus_animalis_(T)* | 0.61 | 0.68 | 0.57 | 0.76 |
| *Lactobacillus_parabuchneri_(T)* | 0.58 | 0.71 | 0.59 | 0.46 |
| *Lactobacillus_florum_(T)* | 0.56 | 0.66 | 0.72 | 0.61 |
| *Lactobacillus_kunkeei_(T)* | 0.55 | 0.62 | 0.55 | 0.61 |
| *Dialister_invisus_(T)* | 0.55 | 0.55 | 0.58 | 0.68 |
| *Streptococcus_salivarius_(T)* | 0.55 | 0.50 | 0.57 | 0.40 |
| *Lactobacillus_coryniformis_(T)* | 0.49 | 0.59 | 0.46 | 0.53 |
| *Aerococcus_viridans* | 0.45 | 0.23 | 0.07 | 0.13 |
| *Lactobacillus_vaccinostercus_(T)* | 0.45 | 0.55 | 0.51 | 0.48 |
| *Lactobacillus_ingluviei_(T)* | 0.41 | 0.42 | 0.28 | 0.34 |
| *Anaerococcus_murdochii_(T)* | 0.40 | 0.54 | 0.30 | 0.26 |
| *Lactobacillus_helveticus_(T)* | 0.36 | 0.00 | 0.33 | 0.31 |
| *Anaerococcus_vaginalis_(T)* | 0.36 | 0.44 | 0.35 | 0.41 |
| *Streptococcus_suis_(T)* | 0.36 | 0.32 | 0.40 | 0.33 |
| *Lactobacillus_paracollinoides_(T)* | 0.33 | 0.30 | 0.29 | 0.35 |
| *Dialister_pneumosintes_(T)* | 0.32 | 0.35 | 0.35 | 0.33 |
| *Lactobacillus_vaginalis_(T)* | 0.30 | 0.31 | 0.22 | 0.46 |
| *Lactobacillus_oeni_(T)* | 0.28 | 0.37 | 0.27 | 0.30 |
| *Mobiluncus_curtisii_(T)* | 0.27 | 0.30 | 0.25 | 0.36 |
| *Lactobacillus_crustorum_(T)* | 0.25 | 0.21 | 0.23 | 0.23 |
| *Lactobacillus_rossiae_(T)* | 0.25 | 0.15 | 0.36 | 0.15 |
| *Ureaplasma_urealyticum_(T)* | 0.25 | 0.30 | 0.28 | 0.30 |
| *Lactobacillus_harbinensis_(T)* | 0.24 | 0.23 | 0.17 | 0.18 |
| *Lactobacillus_acetotolerans_(T)* | 0.24 | 0.22 | 0.22 | 0.28 |
| *Streptococcus_agalactiae_(T)* | 0.24 | 0.28 | 0.28 | 0.29 |
| *Lactobacillus_sunkii_(T)* | 0.23 | 0.30 | 0.21 | 0.25 |
| **Cumulated Percentage Deviation from abundance estimated using full-length 16S sequences** | - | 17.80 | 8.83 | 9.50 |

**Supplementary Table S9H.** Utility of proposed combinatorial approach in obtaining refined taxonomic profiles compared to taxonomic abundance estimates obtained with pair-wise combinations of V-regions. Results in the table pertain to the simulated **Nematode-gut** microbiome dataset (as depicted in Figure 5).

| **Species** | **Abundance (%) estimated with full-length 16S reads** | **Abundance (%) estimated with 10000 V1+V4 paired-end reads** | **Abundance (%) estimated with 10000 V1+V5 paired-end reads** | **Abundance (%) estimated with combinatorial approach using 5000 V1+V4 and 5000 V1+V5 reads** |
| --- | --- | --- | --- | --- |
| *Acinetobacter_baumannii_(T)* | 8.40 | 10.44 | 8.59 | 9.11 |
| *Cellvibrio_vulgaris_(T)* | 7.93 | 0.00 | 10.13 | 9.66 |
| *Cellvibrio_japonicus_(T)* | 6.24 | 8.52 | 7.97 | 7.38 |
| *Cellvibrio_fibrivorans_(T)* | 4.60 | 2.02 | 6.16 | 3.87 |
| *Reyranella_massiliensis* | 3.58 | 5.31 | 5.24 | 4.08 |
| *Pseudoalteromonas_tetraodonis_(T)* | 3.58 | 4.76 | 4.62 | 3.65 |
| *Enhydrobacter_aerosaccus_(T)* | 3.57 | 4.91 | 4.39 | 4.58 |
| *Acinetobacter_calcoaceticus_(T)* | 3.47 | 4.21 | 2.82 | 3.22 |
| *Cellvibrio_mixtus_(T)* | 3.17 | 4.01 | 4.07 | 3.37 |
| *Cellvibrio_sp._E50* | 3.06 | 4.04 | 0.00 | 3.43 |
| *Marinomonas_primoryensis_(T)* | 2.62 | 0.00 | 0.00 | 0.00 |
| *Acinetobacter_lwoffii_(T)* | 2.42 | 2.67 | 2.34 | 2.18 |
| *Escherichia/Shigella_flexneri_(T)* | 2.15 | 0.00 | 0.00 | 0.00 |
| *Escherichia/Shigella_fergusonii_(T)* | 2.15 | 2.89 | 0.00 | 2.37 |
| *Escherichia/Shigella_dysenteriae_(T)* | 2.12 | 2.54 | 2.76 | 2.57 |
| *Staphylococcus_aureus_(T)* | 2.09 | 2.82 | 2.62 | 2.43 |
| *Escherichia/Shigella_albertii_(T)* | 2.01 | 2.72 | 2.87 | 2.30 |
| *Alkanindiges_illinoisensis_(T)* | 1.94 | 2.49 | 2.62 | 2.37 |
| *Pseudomonas_aeruginosa_(T)* | 1.76 | 2.47 | 2.30 | 2.02 |
| *Arcobacter_butzleri_(T)* | 1.74 | 2.14 | 2.16 | 1.66 |
| *Cellvibrio_fulvus_(T)* | 1.74 | 2.27 | 2.32 | 2.04 |
| *Propionibacterium_acnes_(T)* | 1.70 | 2.29 | 2.30 | 2.02 |
| *Cellvibrio_gandavensis_(T)* | 1.63 | 2.27 | 1.75 | 1.92 |
| *Cellvibrio_ostraviensis_(T)* | 1.60 | 2.19 | 2.04 | 2.04 |
| *Marinomonas_arctica_(T)* | 1.56 | 1.94 | 1.79 | 1.94 |
| *Oceanospirillum_maris_(T)* | 1.45 | 2.24 | 2.00 | 1.83 |
| *Peredibacter_starrii_(T)* | 1.39 | 1.84 | 1.79 | 1.54 |
| *Pseudoalteromonas_arctica_(T)* | 1.31 | 0.00 | 0.00 | 0.00 |
| *Delftia_lacustris_(T)* | 1.25 | 0.00 | 0.00 | 0.00 |
| *Oleispira_antarctica_(T)* | 1.17 | 1.54 | 1.54 | 1.52 |
| *Oceanospirillum_beijerinckii_(T)* | 1.08 | 1.50 | 1.47 | 1.44 |
| *Acinetobacter_junii_(T)* | 1.06 | 1.57 | 1.15 | 1.10 |
| *Listonella_anguillarum_(T)* | 1.01 | 0.22 | 0.78 | 0.54 |
| *Brevundimonas_naejangsanensis_(T)* | 1.01 | 1.00 | 1.19 | 1.33 |
| *Pseudoalteromonas_shioyasakiensis* | 0.96 | 0.95 | 1.19 | 0.93 |
| *Oceanospirillum_linum* | 0.89 | 0.72 | 1.10 | 1.22 |
| *Vibrio_cholerae_(T)* | 0.87 | 0.00 | 0.02 | 0.08 |
| *Brevundimonas_diminuta_(T)* | 0.82 | 1.10 | 0.02 | 0.97 |
| *Persicirhabdus_sediminis_(T)* | 0.82 | 1.37 | 1.06 | 0.97 |
| *Leucobacter_chromiiresistens_(T)* | 0.82 | 0.00 | 0.00 | 0.00 |
| *Acinetobacter_radioresistens_(T)* | 0.80 | 1.20 | 0.90 | 0.97 |
| *Brevundimonas_terrae_(T)* | 0.77 | 0.35 | 0.34 | 0.35 |
| *Vibrio_rotiferianus_(T)* | 0.77 | 0.55 | 0.02 | 0.59 |
| *Acinetobacter_guillouiae_(T)* | 0.76 | 0.37 | 0.83 | 0.66 |
| *Leucobacter_tardus_(T)* | 0.76 | 1.02 | 0.85 | 0.95 |
| *Brevundimonas_bullata_(T)* | 0.72 | 1.00 | 0.23 | 0.76 |
| *Leucobacter_komagatae_(T)* | 0.71 | 0.17 | 0.23 | 0.23 |
| *Microbacteriaceae_bacterium_DSM_27064* | 0.67 | 0.52 | 0.21 | 0.56 |
| *Acinetobacter_nectaris_(T)* | 0.66 | 0.85 | 0.62 | 0.67 |
| *Brevundimonas_intermedia_(T)* | 0.64 | 0.00 | 0.62 | 0.59 |
| **Cumulated Percentage Deviation from abundance estimated using full-length 16S sequences** | - | 45.74 | 37.26 | 24.19 |
