# Supplementary figures and images for: Can Targeting Non-Contiguous V-Regions With Paired-End Sequencing Improve 16S rRNA-Based Taxonomic Resolution of Microbiomes?: An In Silico Evaluation
Source: Front Genet. 2019 Jul 12;10:653. doi: 10.3389/fgene.2019.00653 (PMC6640118; doi:10.3389/fgene.2019.00653)

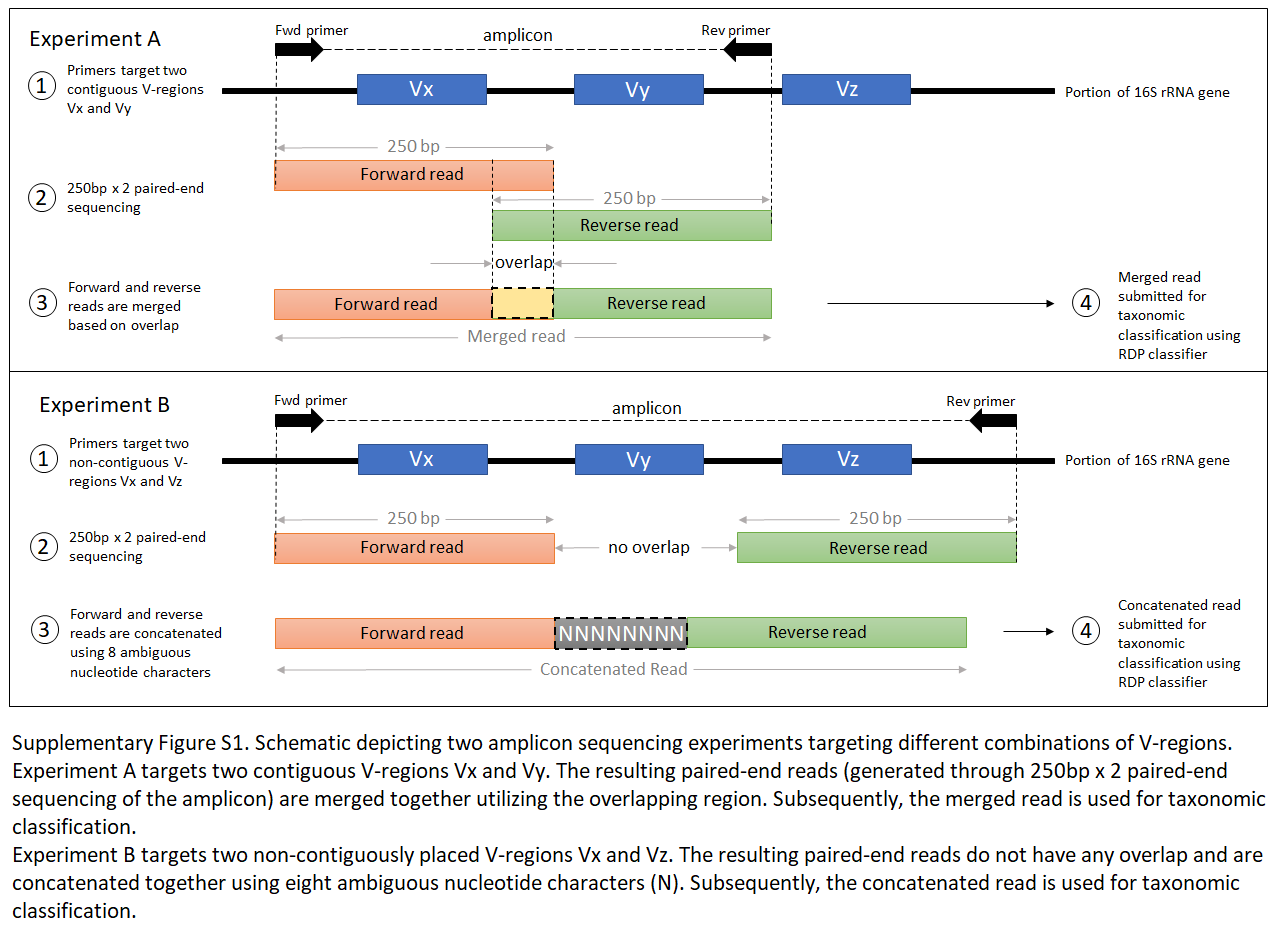

Supplement: Supplementary file 11 [file Image_1.tif]

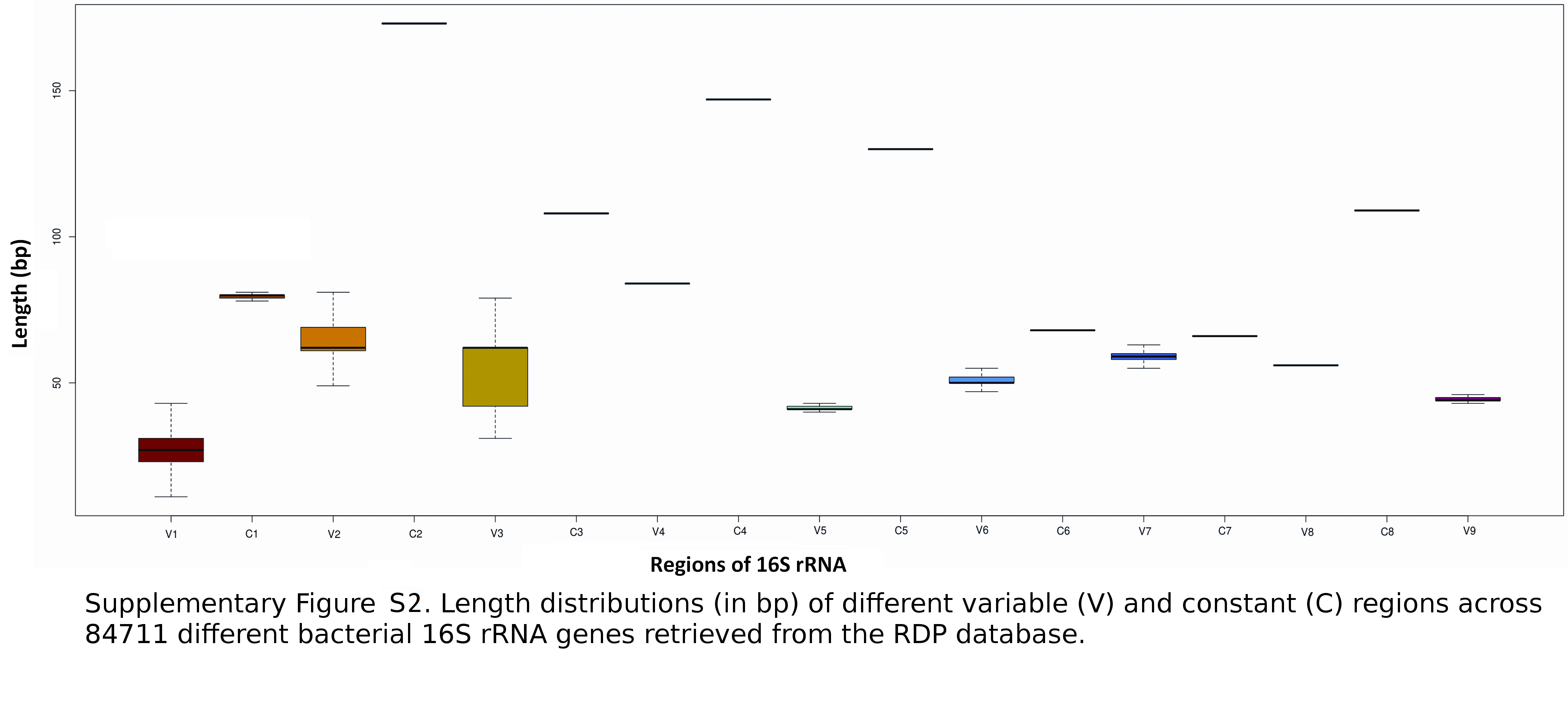

Supplement: Supplementary file 12 [file Image_2.tif]
