# Supplementary material for: Can Targeting Non-Contiguous V-Regions With Paired-End Sequencing Improve 16S rRNA-Based Taxonomic Resolution of Microbiomes?: An In Silico Evaluation
Source: Front Genet. 2019 Jul 12;10:653. doi: 10.3389/fgene.2019.00653 (PMC6640118; doi:10.3389/fgene.2019.00653)

Supplementary Figure S3

(A) A comparison of the paired-end reads generated in the *in silico* experiments with respect to those which may be obtained by using different sets of primers currently available for 16S rRNA amplicon sequencing

Target combination of V-regions	Set of available primers suitable for targeting the given combination	Forward primer sequence (5'-3')	Reverse primer sequence (5'-3')	Percentage (%) of 16S sequences the forward primer is specific to (out of 84,711 full-length sequences from RDP#)	Percentage (%) of 16S sequences the reverse primer is specific to (out of 84,711 full-length sequences from RDP#)	Overlap of read length (%) compared to HMM based extraction of V-region combinations
V1+V4	68F - 798R	TNANACATGCAAGTCGRRCG	AGGATTAGATACCCT	95.24	99.70	77.60
V1+V3	68F - U529R	TNANACATGCAAGTCGRRCG	GCCAGCMGCCGCGGT	95.24	99.45	91.20
V1+V8	68F - 1401R	TNANACATGCAAGTCGRRCG	GGGTCTTGTACACACCG	95.24	99.57	81.56
V1+V7	68F - GM12R	TNANACATGCAAGTCGRRCG	GAGGAAGGTGKGGATGACG	95.24	95.91	88.60
V1+V6	68F - 1061R	TNANACATGCAAGTCGRRCG	GTCGTCAGCTCGTGYYG	95.24	98.66	89.20
V1+V5	68F - 908R	TNANACATGCAAGTCGRRCG	AACTCAAAKGAATTGACG	95.24	99.27	90.60
V1+V9	68F - 1407R	TNANACATGCAAGTCGRRCG	GYACACACCGCCCGTC	95.24	99.47	76.18
V2+V4	pBR-V1.ASF - 798R	AGTGGCGGACGGGTGAGTAA	AGGATTAGATACCCT	95.47	99.70	82.88
V2+V8	pBR-V1.ASF - 1401R	AGTGGCGGACGGGTGAGTAA	GGGTCTTGTACACACCG	95.47	99.57	86.84
V2+V6	pBR-V1.ASF - 1061R	AGTGGCGGACGGGTGAGTAA	GTCGTCAGCTCGTGYYG	95.47	98.66	94.48
V2+V7	pBR-V1.ASF - GM12R	AGTGGCGGACGGGTGAGTAA	GAGGAAGGTGKGGATGACG	95.47	95.91	93.88
V2V3*	pBR-V1.ASF - U529R	AGTGGCGGACGGGTGAGTAA	GCCAGCMGCCGCGGT	95.47	99.45	96.48
V2+V9	pBR-V1.ASF - 1407R	AGTGGCGGACGGGTGAGTAA	GYACACACCGCCCGTC	95.47	99.47	81.46
V1V2*	68F - 338R	TNANACATGCAAGTCGRRCG	ACTCCTACGGGAGGCAGCA	95.24	99.14	79.80
V3+V8	347F - 1401R	GGAGGCAGCAGTRRGGAAT	GGGTCTTGTACACACCG	98.94	99.57	87.56
V3+V7	347F - GM12R	GGAGGCAGCAGTRRGGAAT	GAGGAAGGTGKGGATGACG	98.94	95.91	94.60
V2+V5	pBR-V1.ASF - 908R	AGTGGCGGACGGGTGAGTAA	AACTCAAAKGAATTGACG	95.47	99.27	95.88
V3+V6	347F - 1061R	GGAGGCAGCAGTRRGGAAT	GTCGTCAGCTCGTGYYG	98.94	98.66	95.20
V3+V9	347F - 1407R	GGAGGCAGCAGTRRGGAAT	GYACACACCGCCCGTC	98.94	99.47	82.17
V3+V5	347F - 908R	GGAGGCAGCAGTRRGGAAT	AACTCAAAKGAATTGACG	98.94	99.27	96.60

* contiguous combination

\# *in silico* experiments performed with 84,711 full length sequences from the RDP database which contained all nine V-regions.
(primer specificity evaluated while allowing a maximum mismatch of 2 base pairs)
Result of similar *in silico* experiments for assessing primer specificity performed with all sequences from RDP are presented in Supplementary Table S8.

(B) A comparison of taxonomic profiling accuracy obtained with V1+V5 paired-end reads generated in the *in silico* experiments with respect to those which may be obtained by using 68F-908R primers (currently available for 16S rRNA amplicon sequencing). Results pertaining to simulated host (human) associated microbiomes (as presented in Figure 5) are depicted.

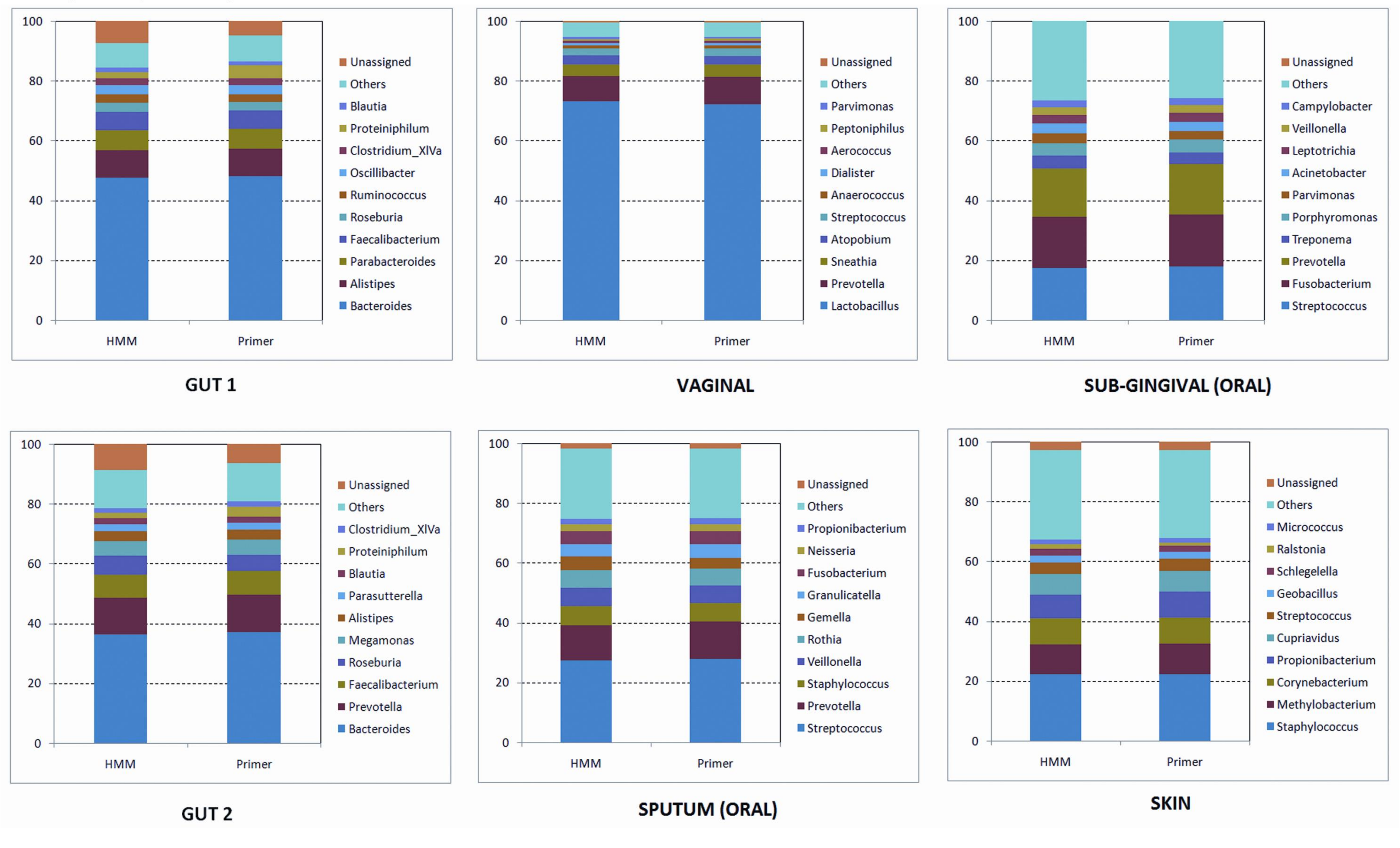

Supplement: Supplementary file 13 [file Image_3.pdf]
